# Age‐related decline of interferon‐gamma responses in macrophage impairs satellite cell proliferation and regeneration

**DOI:** 10.1002/jcsm.12584

**Published:** 2020-07-29

**Authors:** Congcong Zhang, Naixuan Cheng, Bokang Qiao, Fan Zhang, Jian Wu, Chang Liu, Yulin Li, Jie Du

**Affiliations:** ^1^ Beijing Anzhen Hospital Capital Medical University; Key Laboratory of Remodeling‐related Cardiovascular Diseases, Ministry of Education; Beijing Collaborative Innovation Center for Cardiovascular Disorders; Beijing Institute of Heart, Lung and Blood Vessel Diseases Beijing China; ^2^ Section of Physiology and Biochemistry of Sports Capital University of Physical Education and Sports Beijing China

**Keywords:** Aging, Muscle regeneration, Macrophage, Single‐cell RNA sequence, IFN‐γ, CXCL10

## Abstract

**Background:**

Impaired muscle regeneration and increased muscle fibrosis are observed in aged muscle accompanied by progressive loss of muscle mass (sarcopenia). However, the underlying mechanism is still unclear.

**Methods:**

The differentiated expressed genes in young and aged muscles after acute injury by cardiotoxin were identified by RNA‐sequence analysis. Single‐cell RNA‐sequence analysis was used to identify cell clusters and functions in young muscle after acute injury, and flow cytometry analysis and sorting were used to validate the function. The proliferation and differentiation functions of satellite cells were accessed by immunostaining with 5‐ethynyl‐2′‐deoxyuridine and embryonic myosin heavy chain (eMyHC), respectively. Muscle regeneration ability was accessed by histopathological and molecular biological methods.

**Results:**

Gene expression patterns associated with responses to interferon‐gamma (IFN‐γ) (15 genes; false discovery rate < 0.001) were significantly down‐regulated during muscle regeneration in aged mice (*P* = 2.25e−7). CD8^+^ T cells were the main source of increased IFN‐γ after injury, adoptive transfer of wild‐type CD8^+^ T cells to IFN‐γ‐deficient young mice resulted in 78% increase in cross‐sectional areas (CSAs) of regenerated myofibres (*P* < 0.05) and 63% decrease in muscle fibrosis (*P* < 0.05) after injury. Single‐cell RNA‐sequence analysis identified a novel subset of macrophages [named as IFN‐responsive macrophages (IFNRMs)] that specifically expressed IFN‐responsive genes (*Ifit3*, *Isg15*, *Irf7*, etc.) in young mice at 3 days after injury, and the number of this macrophage subset was ~20% lower in aged mice at the same time (*P* < 0.05). IFNRMs secreted cytokine C‐X‐C motif chemokine 10 (CXCL10) that promoted the proliferation and differentiation of satellite cells via its receptor, CXCR3. Intramuscular recombinant CXCL10 treatment in aged mice rejuvenated the proliferation of satellite cells (80% increase in Ki‐67^+^Pax7^+^ cells, *P* < 0.01) and resulted in 27% increase in CSA of regenerated myofibres (*P* < 0.01) and 29% decrease in muscle fibrosis (*P* < 0.05).

**Conclusions:**

Our study indicates that decline in IFN‐γ response in a novel subset of macrophage contributes to satellite cells dysfunctions in aged skeletal muscles and demonstrates that this mechanism can be targeted to restore age‐associated myogenesis.

## Introduction

Aging is associated with a progressive decline in skeletal muscle mass and regenerative capacity. Impaired regeneration process results in weakened muscles strength, which will limit the physical function and affect life quality.^1^, ^2^ The regeneration of skeletal muscle is dependent on the activity of muscle satellite cells (MuSCs) located between the basal lamina and sarcolemma of myofibres. Following acute or chronic damage, quiescent MuSCs (Pax7^+^MyoD^−^) activate into myoblasts (Pax7^+^MyoD^+^), and then the myoblasts undergo proliferation, differentiation, and fusion to form new muscle fibres.^3^, ^4^ As a consequence of aging, the number of MuSCs decreases in aged muscle, and the remaining MuSCs display impaired functions of self‐renewal, activation, proliferation, and differentiation.^5^, ^6^, ^7^ These dysfunctions of MuSCs exhibit an impaired regenerative phenotype, leading to deficient muscle repair following injury. However, the mechanisms responsible for the aging‐associated decline in myogenic capacity of muscle have remained unclear.

In addition to endogenous factors, the functions of MuSCs are also affected by the niche inflammatory micro‐environment.^8^ Circulating Ly6C^hi^ monocytes/macrophages and neutrophils are recruited by injured muscle fibres and infiltrate into skeletal muscle to phagocytize and remove necrotic muscle fibres. The macrophages then convert into Ly6C^low^ pro‐reparative macrophages to secrete growth factors, such as IGF‐1, to promote differentiation and fusion of MuSCs to form new muscle fibres.^9^, ^10^, ^11^ Treg cells, which are accumulated at the late stage of the injury, could inhibit the proinflammatory response and promote the differentiation of pro‐reparative macrophages and skeletal muscle regeneration.^12^ The inflammatory micro‐environments are also altered by aging. It has been reported that amplification of Treg cells was decreased^13^ and differentiation of macrophages was shifted in aged muscle.^14^ Moreover, there are changes of fibro/adipogenic progenitors (FAPs), which is reported to support MuSC myogenic process in young muscle, which lead to abnormal fibrosis and adiposis in aged muscle.^15^, ^16^ Alterations in the levels of cytokines [e.g. fibroblast growth factor‐2,^6^ interleukin‐33,^13^ fibronectin,^17^ Notch ligands,^18^ and WISP1^19^] secreted from niche cells (immune cells or FAPs) have also been reported to disturb MuSC functions and result in impaired muscle regeneration in aged muscle. However, the mechanism for global changes in function of niche cells has not yet been understood completely.

In this study, we unbiased identified that the expression of genes related to interferon‐gamma (IFN‐γ) responses was down‐regulated in aged mice during muscle regeneration after injury. IFN‐γ deficiency impaired muscle regeneration. In response to injury, a novel population of interferon‐responsive macrophages (IFNRMs) was identified by single‐cell RNA‐sequence (RNA‐seq) analysis, and this subset of macrophages was reduced in aged muscle after injury. IFNRMs specifically expressed the secretory cytokine CXCL10 that promoted the proliferation of MuSCs via its receptor, CXCR3. Recombinant CXCL10 treatment rejuvenated MuSC functions and restored muscle regeneration in aged mice.

## Materials and methods

### Animals

Wild‐type (WT), IFN‐γ−/−, and CD8a−/− mice (all C57BL/6 background) were obtained from the National Animal Model Research Center (Nanjing, China) and bred in the animal facility of Beijing An Zhen Hospital affiliated with Capital Medical University. Mice were housed in a specific pathogen‐free environment with constant temperature and humidity and a 12:12 h light–dark cycle. Male mice aged 2, 12, and 24 months were used in experiments. Mouse body weights ranged from 20 to 25 g. Animal experimental protocols were approved by the Animal Subjects Committee of Capital Medical University.

### Muscle regeneration model

Injury‐induced muscle regeneration was established as described previously.^20^ Briefly, tibialis anterior (TA) and gastrocnemius muscles of anaesthetized mice (100 mg/kg of 1% pentobarbital sodium, i.p.) were injected with 30 and 60 μL of 10 μmol/L of cardiotoxin (CTX; Sigma, St. Louis, MO), respectively. At various time points after injury, mice were weighted and sacrificed by cervical dislocation while under anaesthesia. TA muscles were mounted in optimal cutting temperature (OCT) compands, frozen in isopentane that is chilled with liquid nitrogen, and stored at −80°C for pathological analysis. Gastrocnemius muscles were frozen in liquid nitrogen for mRNA and protein extractions.

### Histochemical analyses

Serial, transverse cryosections (7 μm thick) of the mid‐belly region of frozen TA muscles were prepared at −20°C using a CM1950 Frigocut (Leica, Wetzlar, Germany). Cryosections were stored at −80°C until analysis. Picrosirius red staining was used to detect collagen deposition. Immunofluorescence (IF) staining with fluorescein isothiocyanate conjugated wheat germ agglutinin (WGA) (Sigma, 1:100 dilution) was used to analysis of myofibre cross‐sectional area (CSA). For IF staining, the sections were first rehydrated with 1× phosphate‐buffered saline (PBS) and fixed with 4% paraformaldehyde for 10 min and then blocked with 10% bovine serum albumin at room temperature for 30 min. The primary antibodies were diluted in 1× PBS and added for overnight at 4°C. After a washing step with PBS, the corresponding secondary antibodies were added for 1 h at room temperature in dark. The slides were washed with 1× PBS and then mounted with DAPI. All antibodies used for IF staining are listed in Table S4. Four to six images of each muscle section were obtained at ×200 or ×400 magnification as indicated (ECLIPSE 90i; Nikon, Japan) and analysed by NIS‐Elements Br 3.0 software. For analysis, the mean and distribution of myofibre CSA and 200~300 myofibres were measured for one sample.

### Flow cytometric analysis

Flow cytometry was performed as described previously.^20^ TA muscles were dissociated in PBS containing collagenase I (200 U/mL) and Dispase II (2.4 U/mL) at 37°C for 30 min; the single‐cell suspension was filtered, centrifuged, and resuspended in PBS. Antibodies were applied at 4°C for 30 min in the dark, and then the cells were washed and resuspended in PBS. Antibodies used for flow cytometry are listed in Table S4. Expression of surface molecules was analysed by a BD LSRFortessa and associated software (BD FACS Diva Software).

### Western blotting

Proteins from muscle tissues or cells were isolated with protein extraction buffer (Thermo Fisher, Waltham, MA) by incubation on ice for 30 min. After centrifugation at 12 000 rpm for 15 min at 4°C, supernatants were collected. Protein concentrations were determined by a BCA protein assay kit (Thermo Fisher). Western blotting was performed as described previously.^20^ In brief, after blocking in 5% milk powder, membranes were incubated with specific antibodies at 4°C overnight, washed, and then incubated with a secondary antibody (Li‐COR Biosciences, Lincoln, NE) conjugated with IRD800 at room temperature for 1 h in the dark. The membranes were then washed and analysed by the Odyssey software system (Li‐COR). The ratio of the protein change was normalized to GAPDH. Antibodies used in western blotting are listed in Table S4.

### Quantitative real‐time PCR

Total RNA was extracted from gastrocnemius muscles or cells using TRIzol (Invitrogen, Carlsbad, CA) as described previously.^20^ RNA concentrations were measured by a Nanodrop ND‐2000C (Thermo Scientific). Then, RNAs (2 μg) were reverse transcribed using a reverse transcription kit (Promega, Madison, WI). mRNA levels were analysed by quantitative real‐time PCR (qRT‐PCR) performed with 2× SYBR master mix (Takara, Otsu, Shiga) using an iCycler iQ5 (Bio‐Rad, Hercules, CA). Primers are detailed in Table S5. Relative expression levels of genes were calculated based on cycle threshold values using GAPDH as an internal control [gene relative expression = 2(^GAPDH^Ct − ^sample^Ct)].

### Muscle satellite cell isolation and culture

Muscles from hindlimbs of 4‐ to 6‐week‐old C57BL/6 mice were isolated and digested with 2 mL of 2.5 U/mL collagenase type 2 (Sigma) in 10 mM of CaCl_2_ at 37°C for 30 min. After centrifugation and washing with PBS, 1 mL of 1.5 U/mL collagenase D and 2.4 U/mL Dispase II (Roche Applied Science, Indianapolis, IN) were added, and the mixture was incubated at 37°C for 1 h. The muscle slurry was centrifuged at 1500 rpm for 5 min. The pellet was resuspended with DMEM‐F10 medium (Gibco) supplemented with 10% foetal bovine serum and 1% penicillin–streptomycin (PS) and seeded in 5 cm dishes for 2 h to remove fibroblast, and then the unattached cells were transferred to a new Matrigel (BD) precoated 5 cm dishes with. Cells were cultured in 37°C incubator for growth. For satellite cell differentiation, Dulbecco's modified Eagle medium supplemented with 2% horse serum and 1% PS was used as culture medium for 3 days.

### Cell proliferation assays

*In vitro*, to detect the proliferation of cultured satellite cells (SCs) after stimulation, 5‐ethynyl‐2′‐deoxyuridine (EdU) (1 μM) from an EdU kit (RIBOBIO) was added to the culture medium at 4 h before the endpoint of the 24 h coculture, and staining was carried out according to the manufacturer's instructions. The ratio of EdU‐positive cells was analysed by ImageXpress high content image system (Molecular Devices).

### RNA‐sequence analysis

Three to four samples per group were pooled for RNA‐seq. RNA‐seq analysis was carried out using RNA samples converted into individual cDNA libraries using Illumina TruSeq methods employing single reads of 50 base lengths sequenced at 20–30 million read depths using the Illumina HiSeq 2000 instrument. Differential and significant gene expression analyses were carried out using gene‐level RPKM (reads per kilobase of exon model per million mapped reads). Genes were selected using the criteria of an absolute expression level > 1 RPKM in either group with at least a 1.5‐fold change of RPKM and false discovery rate (FDR) of <0.001.

### Single‐cell RNA‐sequencing and analysis

At 3 days after injury, single‐cell suspension of TA muscles was obtained by enzymatic digestion. Minced muscle was digested in PBS containing collagenase I (200 U/mL) and Dispase II (2.4 U/mL) at 37°C for 30 min, filtered, centrifuged, and then resuspended in PBS. The harvested cells were subjected to single‐cell library preparation by 10× Genomics, followed by paired‐end 100 sequencing using the Illumina HiSeq 4000. In total, 5658 cells were sequenced at 57 702 mean reads of sequencing depth per cell. Raw data from 10× Genomics single‐cell RNA‐sequencing (scRNA‐seq) were processed by Cell Ranger v2.1.0. Cellranger mkfastq was used to demultiplex BCL files from HiSeq 4000 into FASTQs for analysis. Cellranger counts were then used to map reads to the reference genome (mm10) on the basis of STAR aligner v2.5.1b43 and generate gene‐cell‐barcode matrices for each sample. The gene‐cell‐barcode matrices from samples were then concatenated. t‐SNE, κ‐means clustering, and differentially expressed gene (DEG) analysis were also performed by Cellranger v2.1.0 or Loupe Cell software.

### Statistical analysis

Values are reported as means ± SEM. Statistical differences between two groups were assessed by the unpaired two‐tailed Student's *t*‐test. For comparison of more than two groups, one‐way ANOVAs were used. For all statistical tests, *P* < 0.05 was considered to indicate a statistically significant difference.

## Results

### Gene expression patterns associated with responses to interferon‐gamma are down‐regulated during muscle regeneration in aged mice

**FIGURE 1:**
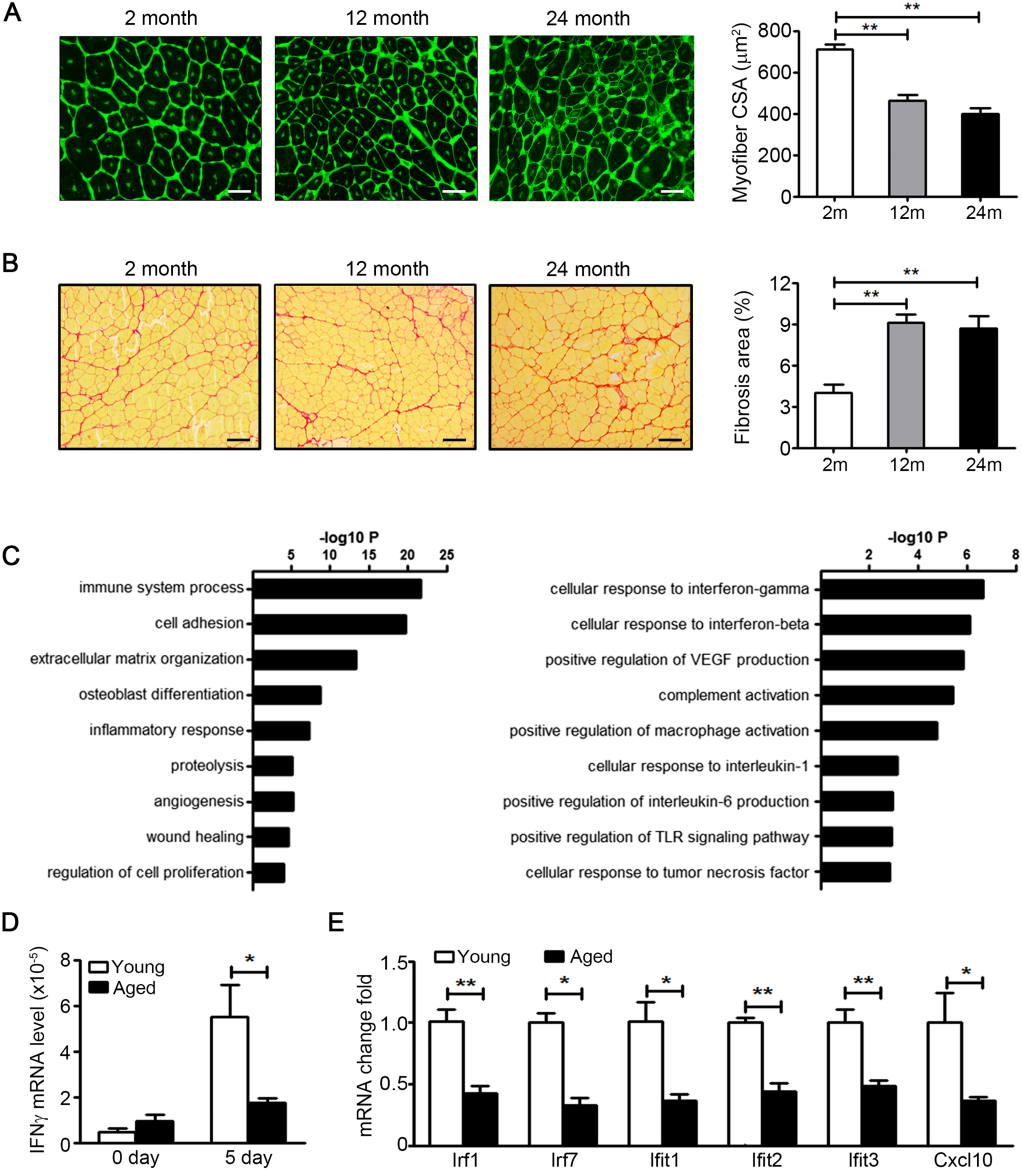
Genes associated with response to interferon‐gamma (IFN‐γ) are down‐regulated during muscle regeneration in aging mice. (A) At day 15 after injury, muscles from 2, 12 and 24 month wild‐type (WT) mice were immunostained with wheat germ agglutinin (WGA) (green, up panel, bars = 50 μm) and Sirus Red (down panel, bars = 100 μm). (B) The mean myofibre cross‐section area (CSA) and ratios of fibrosis area in injured muscles at day 15 after injury were analysed (*n* = 4–6 per group). **P < 0.01, by one‐way ANOVA. (C) The transcription profile of injured 2 and 12 month muscle was analysed by RNA‐seq; DEGs were identified as two‐fold change and FDR of <0.001. The down‐regulated DEGs in 12 month muscle were analysed by ‘GO Biological Process’ categories. (D) The mRNA level of IFN‐γ in muscles from 2 and 24 month WT mice were assessed by real‐time PCR at day 5 after injury (*n* = 4 per group). (E) The mRNA level of IFN‐γ response genes in muscles from 2 and 24 month WT mice were assessed by real‐time PCR at day 5 after injury (*n* = 4 per group). *P < 0.05 and **P < 0.01, by two‐tailed Student's *t*‐test. Data represent the mean ± SEM. DEG, different expressed gene. FDR, false discovery rate.

After acute muscle injury induced by CTX, aged mice (12 or 24 months of age) exhibited impairment of muscle regeneration and increased intramuscular fibrosis (*Figure* 1A and 1B). Flow cytometric analysis revealed more CD45^−^CD31^−^α7‐integrin^+^ myoblasts and less CD45^−^CD31^−^Sca‐1^+^ FAPs in muscle of aged mice than in that of young mice at 5 days after injury (*Figure* S1A, S1B). RNA‐seq was used to identify expressed genes associated with the phenotype of aged muscle. Among the DEGs, mRNAs of 124 genes were up‐regulated and 1059 genes were down‐regulated using a threshold of a two‐fold change and FDR of <0.001 (*Figure* S1C). Gene Ontology functional analysis indicated that genes down‐regulated in aged mice were enriched in the immune system process, cell adhesion, and extracellular matrix organization. Further analysis of expressed genes associated with the immune system process showed enrichment of genes associated with the cellular response to IFN‐γ and interferon‐beta (IFN‐β) (*Figure* 1C, *Figure* S1C). The mRNA level of IFN‐γ was down‐regulated in injured aged muscle (*Figure* 1D). mRNA levels of genes associated with the cellular response to IFN‐γ (*Irf1*, *Irf7*, *Ifit1*, *Ifit2*, *Ifit3*, and *Cxcl10*) in aged muscle were all significantly lower than those in young muscle (*Figure* 1E). The mRNA levels of integrins (*Itgb2*, *Itgb3*, *Itgb5*, and *Itgb7*), which were reported as pro‐regeneration factors in young muscle,^21^ were similar in injured young and aged muscle (*Figure* S1D). These data suggest that a decline in IFN‐γ response may play a role in the impaired regeneration of aged muscle.

### Adoptive transfer of CD8^+^ T cell to interferon‐gamma‐deficient mice rescues impaired muscle regeneration

**FIGURE 2:**
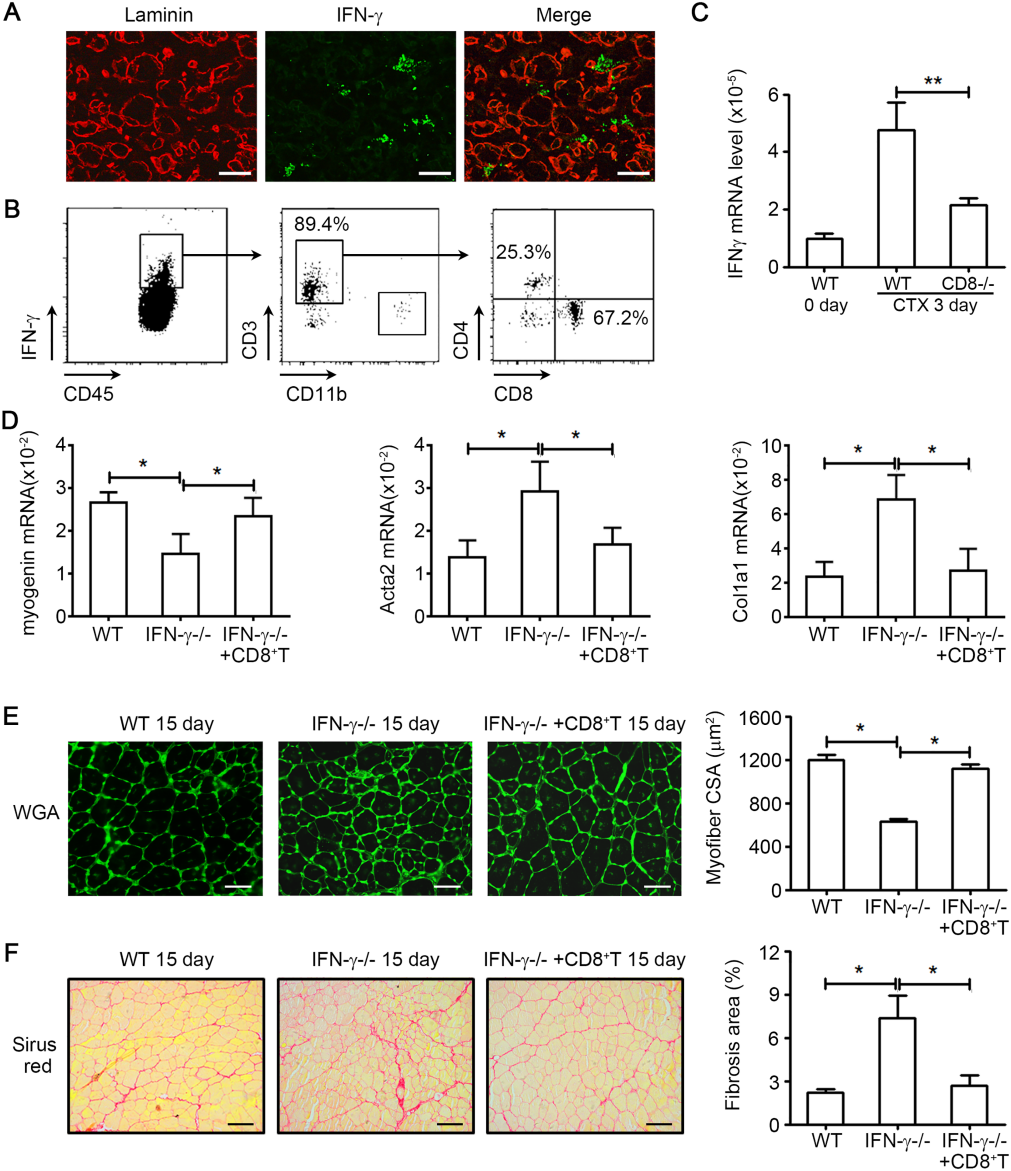
CD8^+^ T cell derived interferon‐gamma (IFN‐γ) is essential to muscle regeneration. (A) Tibialis anterior (TA) muscles at day 5 after injury were co‐immunostained with anti‐IFN‐γ (green) and anti‐laminin (red) antibodies. (B) Intracellular staining of IFN‐γ in CD45^+^ cells and then CD45^+^ IFN‐γ^+^ cells were analysed by CD11b, CD3, CD4, and CD8 staining in the muscle from wild‐type (WT) mice by fluorescence‐activated cell sorting (FACS). (C) The mRNA level of IFN‐γ in muscles from WT and CD8−/− mice were assessed by real‐time PCR at day 5 after injury (*n* = 4 per group). (D) 5 × 10^6^ CD8^+^ T cells sorted from WT spleen were adoptively transferred into IFN‐γ knockout mice after cardiotoxin (CTX) injection immediately. At 5 days after injury, the mRNA level of myogenin, α‐SMA, and Col1α1 in muscles from WT, IFN‐γ−/−, and IFN‐γ−/− with CD8^+^ T mice were assessed by real‐time PCR at day 5 after injury (*n* = 4 per group). (E) At day 15 after injury, muscles from WT, IFN‐γ−/−, and IFN‐γ−/− with CD8^+^ T mice were immunostained with wheat germ agglutinin (WGA) (bars = 50 μm), and the mean myofibre cross‐section area (CSA) was analysed (150~200 myofibre per muscle were examined, *n* = 4 per group). (F) At day 15 after injury, quantification of the ratio of Sirus Red positive fibrosis area in muscle from WT, IFN‐γ−/−, and IFN‐γ−/− with CD8^+^ T mice (bars = 100 μm, *n* = 4 per group). Data represent the mean ± SEM. *P < 0.05 and **P < 0.01, by two‐tailed Student's *t*‐test.

To investigate the role of IFN‐γ in muscle regeneration, we examined IFN‐γ expression levels at various time points in young muscle after CTX‐induced injury. The mRNA and protein levels of IFN‐γ were significantly increased in injured muscle at days 3 and 5 and decreased at day 7 compared with the baseline level (*Figure* S2A, S2B). The mRNA levels of IFN‐γ downstream genes were also increased at 3 days after injury (Table S1). IFN‐γ‐deficient young mice exhibited impairment of muscle regeneration after CTX injury (*Figure* S2C–S2E). To identify cells producing IFN‐γ, dual IF staining of IFN‐γ and laminin was performed. As a result, we found that the IFN‐γ‐positive area was not in the around of laminin‐positive myofibres (*Figure* 2A). Intracellular staining of IFN‐γ in CD45^+^ cells was assessed by flow cytometry. CD8^+^ T cells were the primary cell source of IFN‐γ (~67.2%), CD4^+^ T cells contributed to 25.3% of IFN‐γ‐positive cells, and CD11b^+^ cells produced little IFN‐γ (*Figure* 2B). We next examined the mRNA level of IFN‐γ in CD8‐deficient and WT control young muscles after injury and found that the level of IFN‐γ was lower in CD8‐deficient young muscle than in WT muscle (*Figure* 2C). To confirm the contribution of CD8^+^ T cells to IFN‐γ after injury, CD8^+^ T cells were adoptively transferred to IFN‐γ‐deficient young mice at 1 day after injury. At 5 days after injury, down‐regulated *myogenin* and up‐regulated *Acta2* and *Col1a1* in IFN‐γ‐deficient young muscles (*Figure* S2D) were rescued by adoptive transfer of CD8^+^ T cells (*Figure* 2D). At 15 days after injury, the impaired muscle regeneration and increased muscle fibrosis in IFN‐γ‐deficient young muscles (*Figure* S2E) were also rescued by adoptive transfer of CD8^+^ T cells (*Figure* 2E, 2F). Thus, the primary cell source of IFN‐γ is CD8^+^ T cells in injured muscle.

### Single‐cell sequencing analyses identify a novel subset of interferon‐responsive macrophages in injured muscle

**FIGURE 3:**
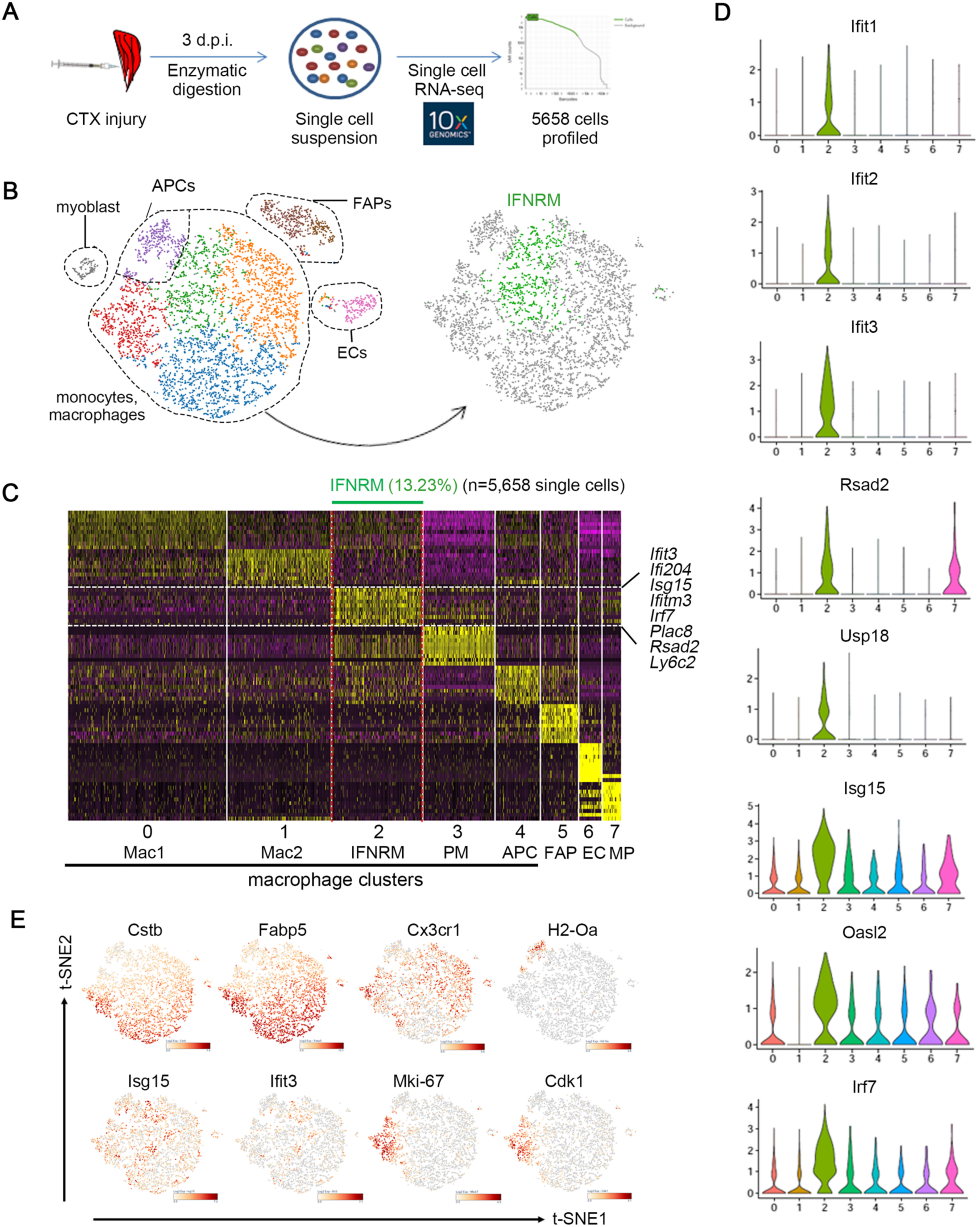
Single‐cell RNA‐seq mapping reveals a unique interferon‐responsive macrophage cluster (IFNRM) in injured young muscle. (A). Cells isolated from injured (3 days) young muscle were used for library preparation of single‐cell RNA sequence, and 5658 quality control‐positive cells was used to further analysis. (B) Data (with each dot representing a single cell) are displayed as colour‐coded clusters on a t‐SNE plot. In the macrophage sub‐cluster, the IFNRM cluster shown in green is defined on the basis that most of the top differentiation expressed marker genes of this cluster were IRGs. (C) Heatmap plotting the top 10 differentiation expressed marker genes for each macrophage sub‐cluster (*y* axis) versus single cells grouped by cluster (*x* axis). The IFNRM population is indicated at the top of the heat map, and selected marker genes are annotated on the right (purple and yellow indicate low and high expression levels, respectively). (D) Violin plots depicting the expression probability distribution for each single‐cell cluster. Eight IRGs are shown. (E) t‐SNE visualization overlaid with the expression of Cstb, Fabp5, Cx3cr1, H2‐Oa, *Isg15*, *Ifit3*, Mki‐67, Cdk1. t‐SNE, t‐distributed stochastic neighbour embedding. IRGs, interferon‐responsive genes.

To gain a mechanistic insight into the IFN‐γ‐associated response in muscle regeneration, we performed single‐cell RNA‐seq profiling of cells isolated from WT young muscles at day 3 after CTX‐induced injury (*Figure* 3A). Unbiased clustering of the cells from injured muscle identified eight cell clusters including five subtypes of macrophages and antigen presenting cells (APCs), myoblasts, FAPs, and endothelial cells (*Figure* 3B, Table S4). These cell clusters were verified by specific cell markers *H2‐Aa* (encoding MHCII) for APCs, *Adgre1* (encoding F4/80) for macrophages, *Itga7* and *Myod1* for myoblasts, *Tie1* and *Pecam1* (encoding CD31) for endothelial cells, and *Ly6a* (encoding Sca‐1) and *Pdgfra* for FAPs (*Figure* S3). A unique population of IFN‐responsive macrophages (IFNRMs) was identified among the macrophage clusters (*Figure* 3B, *Figure* S4). IFNRMs were identified as cells with specific expression of IFN‐responsive genes (IRGs) *Ifit3*, *Ifi204*, *Isg15*, *Ifitm3*, *Irf7*, *Plac8*, *Rsad2*, and *Ly6c2* (*Figure* 3C). Other IRGs were also highly expressed in IFNRM (*Figure* 3C). Based on the function analysis of marker gene expression, the IFNRM was different from other macrophage clusters. The Mac1 cluster expressed a high level of lysosome‐associated genes (*Cstb*, *Ctsl*, etc.) and lipid metabolism‐associated genes (*Fabp5*, *Cd36*, etc.); the Mac2 cluster expressed a high level of *Cx3cr1*, which was reported as a cell marker of anti‐inflammatory and pro‐myogenesis macrophages; the proliferating macrophage clusters expressed a high level of cell cycle‐associated genes (*Mki‐67*, *Cdk1*, etc.); and the APC cluster expressed a high level of antigen presentation‐associated genes (*H2‐Oa*, *H2‐Db2*, etc.) (*Figure* 3E, Table S2). Thus, there is a unique IFNRM cluster in injured young muscle.

### Interferon‐gamma stimulates interferon‐responsive macrophages to produce cytokine CXCL10

**FIGURE 4:**
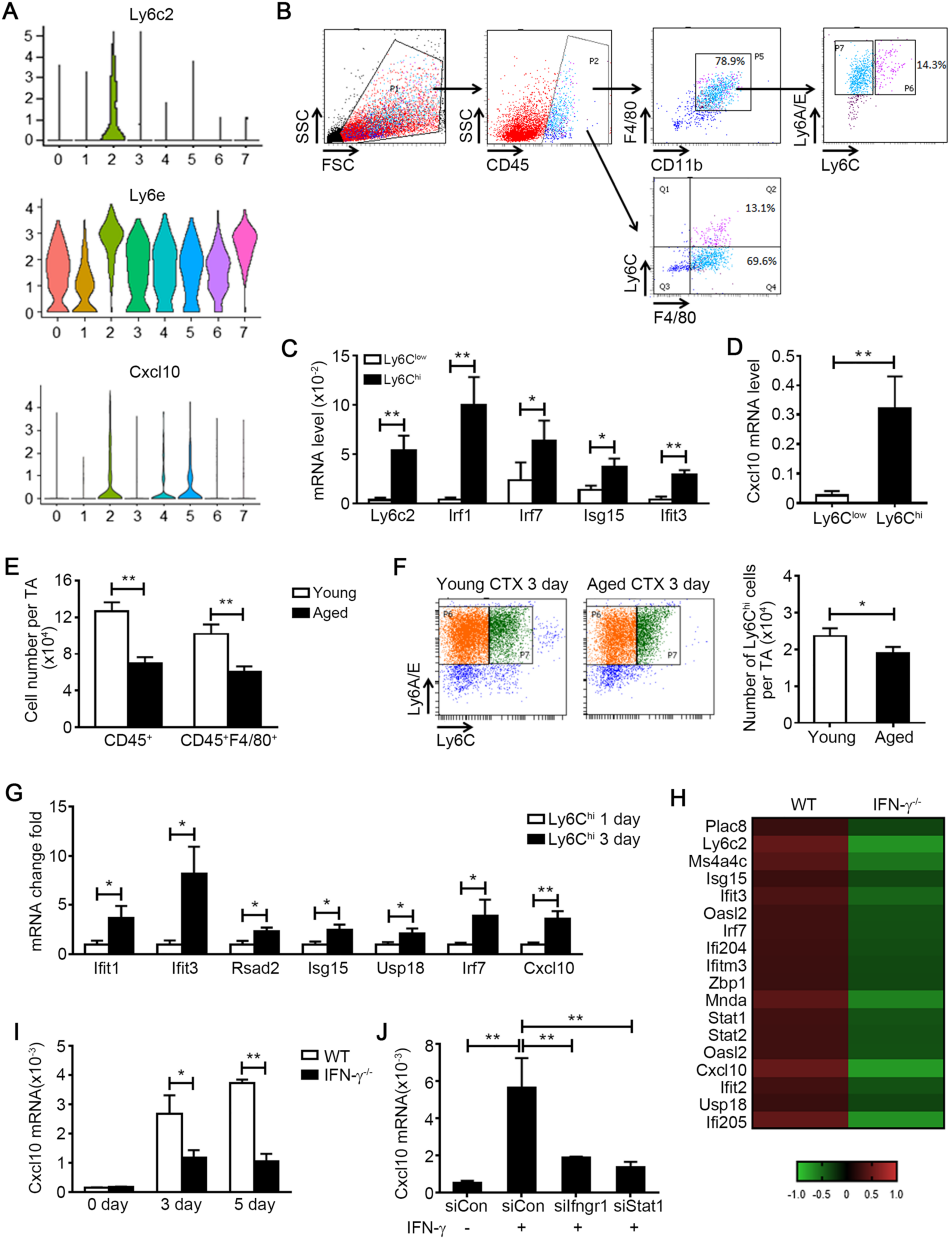
Interferon‐gamma (IFN‐γ) stimulates interferon‐responsive macrophage cluster (IFNRM) to produce a secretory cytokine CXCL10 specifically. (A) Violin plots depicting the expression distribution of membrane protein gene Ly6c2, Ly6e, and secreted protein gene *Cxcl10* in each cell cluster. (B) Representation of gating strategy for sorting the Ly6E^+^Ly6C^hi^ and Ly6E^+^Ly6C^low^ macrophage from young muscle at 1 and 3 days after injury. (C) The expression levels of Ly6c2 and other IRGs (*Irf1*, *Irf7*, *Isg15*, and *Ifit3*) in sorted cells were analysed by real‐time PCR (*n* = 3 per group). (D) The expression levels of *Cxcl10* in sorted cells were analysed by real‐time PCR (*n* = 3 per group). (E and F) The number of CD45^+^ leucocytes, CD45^+^F4/80^+^ macrophages (E) and Ly6E^+^Ly6C^hi^ IFNRM (F) per tibialis anterior (TA) muscle from young and aged mouse were accessed by flow cytometry at 3 days after injury (*n* = 4 per group). (G) The expression levels of (IFNRM) signature genes (*Ifit3*, *Ifit1*, *Rsad2*, *Isg15*, *Usp18*, *Irf7*, and *Cxcl10*) in Ly6C^hi^ macrophages sorted from muscle at 1 day and 3 days after injury were accessed by real‐time PCR (*n* = 3 per group). (H) F4/80^+^ macrophages were sorted from WT and IFN‐γ−/− muscle at 3 days after injury. Then the transcription profile was analysed by RNA‐seq. Heatmap demonstrates the normalized expression level of the top differentiation expressed marker genes of IFNRM cluster in WT and IFN‐γ−/− macrophages. The gene expression levels were normalized against mean expression values of each gene in all samples. (I) the mRNA level of *Cxcl10* in WT and IFN‐γ−/− muscle at 0, 3, and 5 days after injury was accessed by real‐time PCR (*n* = 4 per group). (J) Bone marrow derived macrophage was pre‐treated with control siRNA, Ifngr1 siRNA, or Stat1 siRNA, and then recombinant IFN‐g was added to the culture medium for 24 h. The mRNA level of *Cxcl10* in macrophages was accessed by real‐time PCR (*n* = 4 per group). Data represent the mean ± SEM. *P < 0.05 and **P < 0.01, by two‐tailed Student's *t*‐test.

To explore the function of IFNRMs, we analysed the most characteristic genes according to subcellular locations and found that Ly6C and Ly6E were the most specific membrane proteins and CXCL10 was the most expressed secretory protein (Table S3). Violin diagram analysis showed that Ly6c2 was presented in the IFNRM cluster only, Ly6e was presented in all cell clusters and highly expressed in the IFNRM cluster, CXCL10 was presented in three macrophage clusters, and the highest expression of CXCL10 was in the IFNRM cluster (*Figure* 4A). Next, we isolated IFNRMs from injured young muscles by fluorescence‐activated cell sorting on the basis of the expression of Ly6C and Ly6E. After gating CD45^+^CD11b^+^F4/80^+^ macrophages, two subsets of cells were sorted: Ly6E^+^Ly6C^low^ and Ly6E^+^Ly6C^hi^ cells (*Figure* 4B). The presence of the IFNRM gene signature was confirmed by qPCR in sorted macrophages. Indeed, the Ly6E^+^Ly6C^hi^ subset was characterized by expression of *Ly6c2* and higher expression levels of IRGs (*Irf1*, *Irf7*, *Isg15*, and *Ifit3*) (*Figure* 4C). *Cxcl10* expression was higher in Ly6E^+^Ly6C^hi^ cells than in Ly6E^+^Ly6C^low^ cells (*Figure* 4D). We also examined this Ly6E^+^Ly6C^hi^ IFNRM macrophages in aged muscle post injury, and we found that the number of CD45^+^ leucocyte and CD45^+^F4/80^+^ macrophages was decreased in aged muscle at 3 days after injury (*Figure* 4E). In macrophages population, there were Ly6E^+^Ly6C^hi^ macrophages in aged muscle, but the number of Ly6E^+^Ly6C^hi^ macrophages was significantly decreased in aged muscle (*Figure* 4F). This result was consistent with the down‐regulation of genes associated with IFN‐γ response pathway in aged muscle.

It is known that Ly6C^hi^ macrophages are recruited from circulation at 24 h after injury,^9^ but the transcription profile is altered in Ly6C^hi^ macrophages at various time points.^22^, ^23^ Therefore, we sorted CD45^+^CD11b^+^F4/80^+^Ly6C^hi^ macrophages from young muscles at 1 and 3 days after injury and compared the expression of IFNRM signature genes in these cells. In addition to other IFNRM signature genes (*Ifit3*, *Ifit1*, *Rsad2*, *Isg15*, and *Usp18*), the expression of *Cxcl10* was higher in macrophages at day 3 than in macrophages at day 1 (*Figure* 4G). Macrophages from WT and IFN‐γ knockout young muscles were sorted at day 3 after injury and were analysed by RNA‐seq. The IFNRM signature genes were down‐regulated in IFN‐γ knockout macrophages (*Figure* 4H). The increase of *Cxcl10* expression paralleled the increase of IFN‐γ in muscle after injury. When IFN‐γ was deficient, CXCL10 expression was also decreased (*Figure* 4I). IFN‐γ stimulated the macrophages to increase expression of CXCL10 *in vitro*, which was blocked by IFNγR1 or STAT1 inhibition using siRNAs (*Figure* 4J). Thus, IFN‐γ activates IFNRMs to produce secretory cytokine CXCL10.

### CXCL10 promotes the proliferation of muscle satellite cells via CXCR3

**FIGURE 5:**
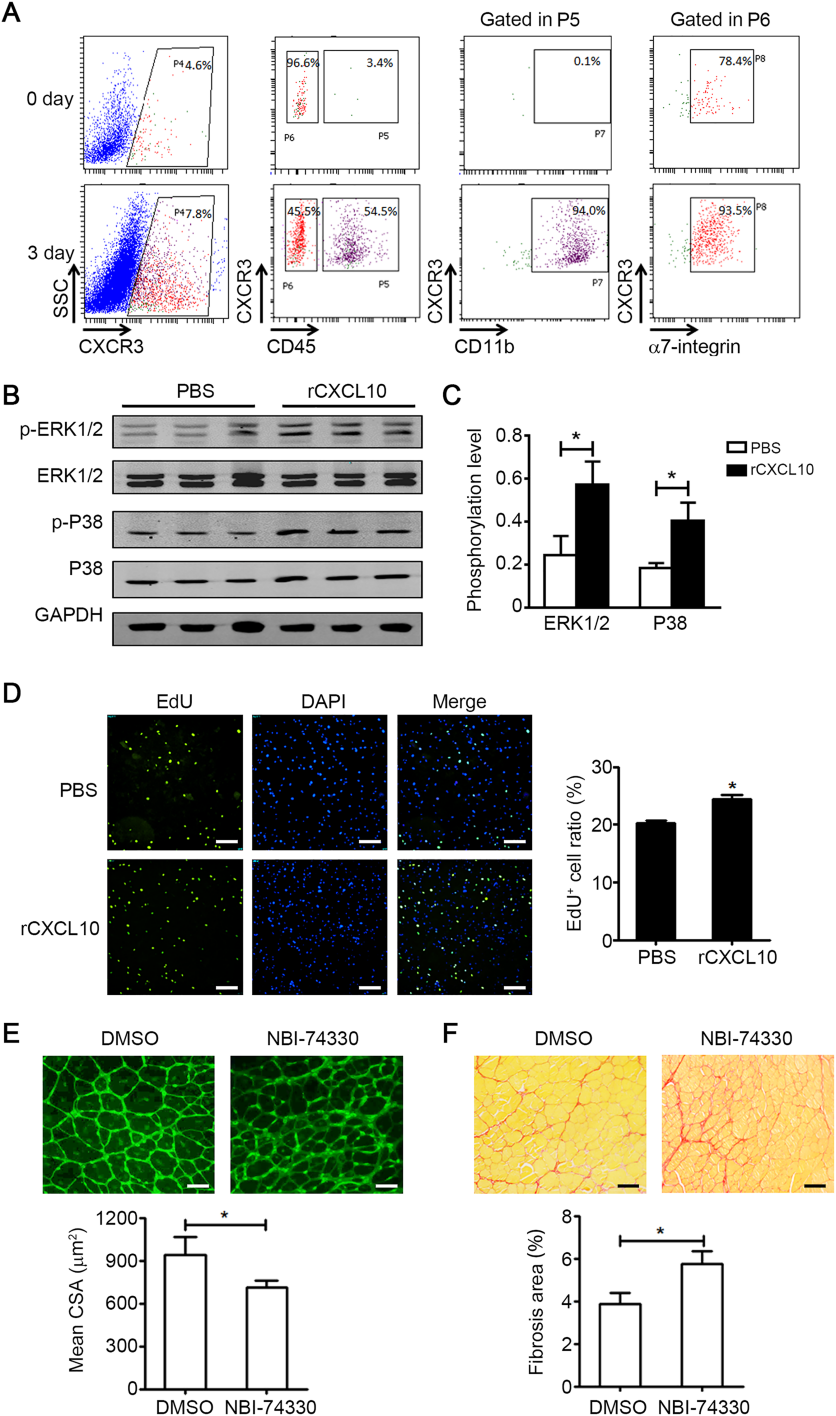
CXCL10 promotes the proliferation and differentiation of muscle satellite cells (MuSCs). (A) Flow cytometry analysis of CXCR3^+^ cells in young muscle at day 0 and day 3 after injury. (B and C) Primary myoblasts were treated with either phosphate‐buffered saline (PBS) or rCXCL10 (20 ng/mL) for 30 min; and phospho‐ERK1/2 (p‐ERK1/2), phospho‐MAPKp38 (p‐P38), total ERK1/2, and total MAPKp38 (P38) protein levels were quantified by western blot (B) and normalized to GAPDH (C) (*n* = 3 per group). (D) Quantification of the ratio of EdU^+^ primary myoblasts cultured for 24 h in medium with either PBS or rCXCL10 (20 ng/mL) (bars = 100 μm, *n* = 6 per group). (E) CXCR3 antagonist NBI‐74330 (100 mg/kg) or DMSO was administered i.v. at 1 and 3 days after injury. The mean myofibre cross‐section area (CSA) was accessed by wheat germ agglutinin (WGA) staining at 15 days after injury (bars = 50 μm, 150~200 myofibre per muscle were examined, *n* = 4 per group). (F) At day 15 after injury, the ratio of Sirus Red positive fibrosis area in muscle from muscle with NBI‐74330 or DMSO treatment (bars = 100 μm, *n* = 4 per group) was quantified. Data represent the mean ± SEM. *P < 0.05, by two‐tailed Student's *t*‐test.

Despite its chemotactic nature, CXCL10 is also reported to be a cytokine associated with cell proliferation, apoptosis, differentiation, and angiogenesis.^24^, ^25^, ^26^, ^27^ The receptor of CXCL10 is CXCR3, a G protein‐coupled receptor expressed mainly in immune cells and other somatic cells. In uninjured young muscle, CXCR3 was expressed mostly in CD45^−^CD31^−^α7‐integrin^+^ MuSCs (75.7%) as assessed by flow cytometry. In young muscles at 3 days after injury, 45.5% of CXCR3^+^ cells were CD45^−^CD31^−^α7‐integrin^+^ MuSCs, and 54.5% of CXCR3^+^ cells were CD45^+^ CD11b^+^ macrophages (*Figure* 5A). The macrophages in muscle were recruited from circulation by other chemokines (e.g. CCL2 and CCL5) at 24 h after injury. Therefore, we investigated the role of the CXCL10–CXCR3 axis in myogenesis. Recombinant CXCL10 (rCXCL10) treatment (20 ng/mL) of primary myoblasts induced phosphorylation of ERK1/2 and MAPKp38 *in vitro* (*Figure* 5B, 5C). ERK1/2 and MAPKp38 pathways have been implicated in proliferation and differentiation of MuSCs.^28^, ^29^ Addition of rCXCL10 (20 ng/mL) to the culture medium could increase the proliferation of primary myoblasts (*Figure* 5D) and promote the differentiation of primary myoblasts *in vitro* (*Figure* S5). We next used a CXCR3 antagonist (NBI‐74330) to examine whether blocking the CXCL10‐CXCR3 axis affected muscle regeneration in young mice *in vivo*. To this end, mice were treated by injections of either NBI‐74330 or DMSO during muscle injury. When the CXCR3 antagonist was applied, the CSA of regenerating myofibres was decreased (*Figure* 5F) and the accompanying fibrosis was increased (*Figure* 5G). These data indicate that by stimulating pathways involved in proliferation and myogenic commitment, CXCL10 positively influences muscle regeneration processes in young mice.

### Recombinant CXCL10 treatment rejuvenates muscle satellite cell functions and restores muscle regeneration in aged mice

**FIGURE 6:**
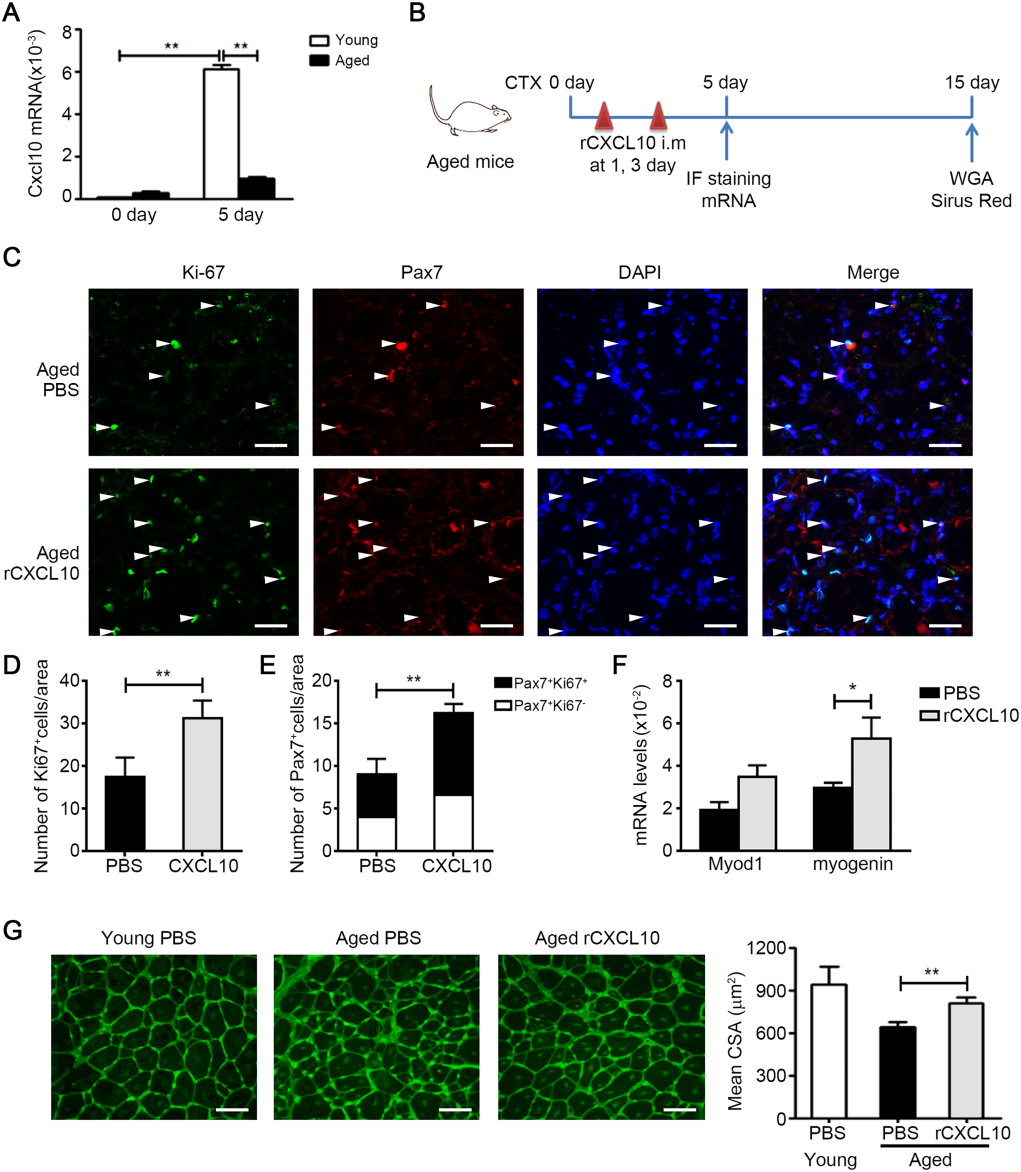
Recombinant CXCL10 treatment rejuvenates muscle satellite cell (MuSC) function and restores muscle regeneration in aged mice. (A) The mRNA level of CXCL10 in muscles from young and aged (24 month) wild‐type (WT) mice were assessed by real‐time PCR at day 5 after injury (*n* = 4 per group). (B) Experimental overview of phosphate‐buffered saline (PBS) or rCXCL10 [1 μg per tibialis anterior (TA) muscle] treatment in aged mouse after injury. (C) Representative images of Ki‐67(green) and Pax7 (red) immunostainings of cross sections of PBS or rCXCL10‐treated aged muscles at 4 days after injury. Arrow indicates Pax7^+^Ki67^+^ MuSCs (bars = 50 μm). (D and E) Quantification of the number of Ki‐67^+^ cells (D) and Pax7^+^/Ki‐67^+^ and Pax7^+^/Ki‐67^−^ MuSCs (E) in PBS or rCXCL10‐treated aged muscles at 5 days after injury (*n* = 3 per group). (F) The mRNA levels of Myod1, myogenin in PBS, or rCXCL10‐treated aged muscles at 5 days after injury was accessed by real‐time PCR (*n* = 4 per group). (G) The mean myofibre CSA of PBS or rCXCL10‐treated aged muscles was accessed by wheat germ agglutinin (WGA) staining at 15 days after injury (bars = 50 μm, 150~200 myofibre per muscle were examined, *n* = 4 per group). Data represent the mean ± SEM. *P < 0.05 and **P < 0.01, by two‐tailed Student's *t*‐test.

The up‐regulation of CXCL10 mRNA was blunted in regenerating muscles of aged mice (*Figure* 6A). We next determined whether restoration of CXCL10 levels ameliorate the impaired regeneration of aged muscles. To this end, aged mice were treated by muscle injections of either rCXCL10 (1ug per TA muscle) or PBS at 1 and 3 days after injury (*Figure* 6B). Firstly, we examined the macrophage population change following administration of rCXCL10 or PBS in young and aged muscle after injury. We found that the number of CD45^+^ leucocytes and CD45^+^F4/80^+^ macrophages was decreased in aged muscle at 3 days after injury. However, the number of CD45^+^F4/80^+^ macrophages was not changed after administration of rCXCL10 in aged the muscle (*Figure* S6A), and there was also no difference in the ratio of Ly6C^hi^ and Ly6C^low^ macrophages in rCXCL10 or PBS‐treated aged muscle (*Figure* S6B). Secondly, we examined the regeneration phenotypes. In muscle cross sections of aged mice treated with rCXCL10, the number of proliferating cells expressing the marker Ki67 was significantly increased compared with the PBS control at 4 days after injury (*Figure* 6C, 6D). Moreover, the numbers of MuSCs and Ki‐67‐positive MuSCs were both significantly increased in rCXCL10‐treated aged muscle (*Figure* 6C, 6E). The mRNA levels of *Myod1* and *myogenin* were significantly higher in rCXCL10‐treated aged muscle than in PBS‐treated muscle at 5 days after CTX injury (*Figure* 6F). rCXCL10 treatment also improved the overall architecture of aged regenerating muscles (*Figure* 6G) and resulted in significantly larger regenerating fibres indicative of efficient regeneration (*Figure* 6H). We also examined the expression levels of Trim63 and Fbxo32, two genes related to protein synthesis and degradation,^30^ in aged muscles with rCXCL10 or PBS‐treatment, and we found that there was no significant difference in the expression of Trim63 and Fbxo32 between rCXCL10‐treated and PBS‐treated aged muscles (*Figure* S6C). These data indicated that there were no changes in muscle protein synthesis or degradation during the recovery of injured satellite cells. In addition, expression of fibrosis‐associated genes *Acta2* and *Col1a1* was reduced by rCXCL10 treatment in aged muscle at 5 days after injury (*Figure* S6D). The Sirus Red positive fibrotic area was reduced at 15 days after injury (*Figure* S6E). Thus, exogenous CXCL10 treatment rejuvenates the myogenic functions of aged MuSCs and restores muscle repair of aged mice.

## Discussion

With the increase of age, the regenerative capacity of muscle declines and abnormal extracellular matrix deposition increases in the muscle interstitial, which influence movement ability. However, the underlying mechanism is still unclear. In this study, a unique cell cluster of IFNRMs in injured young muscle was identified by scRNA‐seq. IFN‐responsive macrophages produced CXCL10 that promoted the proliferation of MuSCs. Furthermore, administration of rCXCL10 rejuvenated MuSCs functions and restored muscle regeneration in aged mice.

It has been reported that the activity of CD8^+^ cytotoxic T cells diminishes in elderly persons after viral infection.^31^ Moreover, lymphocytes from old mice produce markedly lower amounts of IFN‐γ relative to young mice.^32^ In this study, we explored mechanism wherein by influencing muscle regeneration and fibrosis during aging, we found that the IFN‐γ response pathway was down‐regulated in aged muscle, compared with young muscle, after injury. The primary cellular source of IFN‐γ was CD8^+^ T cells. When IFN‐γ or CD8^+^ T cells are deficient, the regeneration ability of young muscle declines and muscle fibrosis increases after acute injury.^33^, ^34^

Identification of reduced IFN‐γ production as a key event for impaired muscle regeneration in aging is supported by its known function in muscle, and the role of IFN‐γ in skeletal muscle regeneration is bilateral. IFN‐γ significantly increases in young muscles at the late stage of injury, and neutralizing antibody blockade or gene knockout inhibits skeletal muscle regeneration.^33^ Supplementation of IFN‐γ reduces fibrosis and improves functions of young muscle in a laceration model.^35^ However, in the mdx muscle model with the presence of a persistent inflammatory response, IFN‐γ inhibits the regeneration process by inhibiting the expression of myogenin.^36^ Studies have shown that Treg cells accumulating in muscles at the late stage of the injury inhibit the production of IFN‐γ, thereby suppressing the proinflammatory response and promoting the differentiation of pro‐reparative macrophages and skeletal muscle regeneration.^12^ Catabolic conditions, such as chronic kidney disease (CKD), chronic heart failure (CHF), and diabetes, were also known as chronic inflammatory states. Increased serum levels of tumour necrosis factor α, interleukin (IL)‐6, and IL‐1β may be associated with muscle waste by enhancing muscle protein degradation.^37^ It was also reported that patients with CKD or CHF often have increased angiotensin II (Ang II) levels, and Ang II could deplete the basal pool of satellite cells and inhibit their proliferation after injury, which resulted in decreased number of satellite cells.^38^ There was no direct relationship between the muscle waste under catabolic condition and IFN‐γ.

In this study, we identified a novel macrophage cluster that determines muscle regeneration capacity between young and aged muscle. The Mac1 cluster expressed a high level of lysosome‐associated genes and lipid metabolism‐associated genes, which was consistent with the main function of macrophages to clear the necrotic myofibre by phagocytosis.^9^ The Cx3cr1 high expression Mac2 cluster and proliferating macrophage cluster were consistent with the report that macrophage switched to CX3CR1^hi^ macrophage and acquired the proliferation ability after phagocytosis.^9^ The novel clusters of macrophages expressed IRGs (*Ifit3*, *Ifi204*, *Isg15*, *Ifitm3*, *Irf7*, *Plac8*, *Rsad2*, and *Ly6c2*), and we named these cells as IFNRMs. The IFNRM cluster was different from other macrophage clusters. *Ly6c2* mRNA was specifically expressed in IFNRM at 3 days after injury. *Ly6c2* encoded protein Ly6C is previously considered to be a cell marker to distinguish the Ly6C^hi^ inflammatory macrophages and Ly6C^low^ repair macrophages.^39^ The function of IFN‐γ is mainly mediated by activation of the relevant intracellular signalling pathways by IFNgR1 and IFNgR2 receptor complexes, and macrophages express both receptors.^40^ IFN‐γ stimulation activates STAT1‐IRF signalling pathways in macrophages, thereby promoting the expression of IRGs.^41^ We showed that the expression levels of IFNRMs specifically expressed genes in macrophages from IFN‐γ knockout young mice were significantly down‐regulated, demonstrating that the differentiation of IFNRMs from circulation‐derived Ly6C^hi^ macrophages was dependent on IFN‐γ.

The integrins were increased in young muscles after CTX‐induced injury, and an improvement in integrin‐β3 function could stimulate muscle regeneration.^21^ We compared the expression change trends of integrin‐β3 and IFN‐γ after muscle injury, and we found that the dynamic changes of integrin‐β3 were different from those of IFN‐γ. The integrin‐β3 was increased at 5 h after injury, and this response persisted for up to 6 days after injury, while the mRNA levels of IFN‐γ were significantly increased in injured muscle at days 3 and 5 and decreased at day 7, as compared with the baseline level. In addition, we analysed the mRNA expression level of some integrins in young and aged muscle after injury at 5 days by real‐time PCR. We found that the mRNA levels of *Itgb2*, *Itgb3*, *Itgb5*, and *Itgb7* were similar in young and aged muscle, which indicated that integrin‐responsive macrophage is different from IFN‐γ‐responsive macrophage. The main function of integrins was regulating the infiltration and polarization of macrophage,^21^ while the main function of IFNRM was promoting the proliferation and differentiation of MuSCs.

Among the genes specifically expressed by IFNRMs, *Cxcl10* encoded protein CXCL10 was the most highly expressed secreted protein. CXCL10 is originally isolated in a screen for genes induced by IFN‐γ.^42^ In this study, we have shown that the increase of CXCL10 expression paralleled with the increase of IFN‐γ in young muscles after injury, CXCL10 was significantly down‐regulated in injured IFN‐γ knockout young muscle and injured WT aged muscles with decreased IFN‐γ, which indicated the importance of IFN‐γ in up‐regulation CXCL10 production after injury.

CXCL10 is a chemokine of the CXC subfamily, whose main function is stimulation of monocyte, natural killer cell, and T‐cell migration.^43^ Recently, a series of studies showed that CXCL10 also could play roles in regulating cell proliferation, apoptosis, differentiation, and angiogenesis.^24^, ^25^, ^26^, ^27^ Our finding that expression of CXCL10 was accompanied by an increase of IFN‐γ at the late stage after injury, while the inflammatory cells were recruited from circulation mainly at the early stage after injury, hints a novel role of CXCL10 in skeletal muscle repair. We found that MuSCs also expressed the receptor of CXCL10 and CXCR3. CXCL10 promoted the proliferation and differentiation of MuSCs via the CXCR3 *in vitro*. These pro‐proliferation effects of CXCL10 have been reported to contribute to various regenerative processes.^44^, ^45^, ^46^ CXCL10 promoted the proliferation of mouse MuSCs by activating ERK1/2 and MAPKp38 pathways *in vitro*. ERK1/2 and MAPKp38 pathways have been reported to participate in the proliferation and differentiation of MuSCs.^28^, ^29^ CXCL10 and CXCL9 share the same receptor, CXCR3. In our study, complete blockade of CXCR3 activation by a CXCR3 antagonist resulted in impaired skeletal muscle regeneration in young mice, demonstrating the necessity of activation of the CXCR3 signalling pathway for regeneration. When CXCL10 is knocked out, CXCL9 could activate CXCR3 to facilitate the process of skeletal muscle regeneration in young mice as a compensatory mechanism.^47^ In this study, we found that the expression of CXCL10 and CXCL9 was significantly down‐regulated simultaneously after injury in aged muscle. Under this condition, replenishment by recombinant CXCL10 after injury in aged mice could significantly increase the proliferation of MuSCs and improve muscle regeneration, which indicated the critical role of IFNRM derived CXCL10 in aged muscle.

In summary, the current study demonstrates that a defect of the IFN‐γ‐associated response in aged muscle plays a critical role in MuSCs dysfunctions. The IFN‐γ‐associated signalling pathway appears to be a promising avenue to explore attempts to overcome these problems.

## Conflict of Interest

The authors declare no competing financial interests.

## Supporting information

Figure S1. IFN‐response pathway was downregulated in aged muscle after injury.
Figure S1. IFN‐response pathway was downregulated in aged muscle after injury.
Figure S3. Distribution of marker genes in cell cluster of muscle at 3 day after injury.
Figure S4. The flow scheme of identify the IFNRM cluster from monocyte/macrophage cluster by Loupe Cell software.
Figure S5. Recombinant CXCL10 promotes the differentiation of primary MuSCs in vitro.
Figure S6. Recombinant CXCL10 treatment decreases the muscle fibrosis during regeneration in aged mice.
Table S1. Antibody list.
Table S2. Oligonucleotide sequences for quantitative realtime‐PCR.
Table S3. Expression levels of IFN‐responsive genes in muscle from 0 day and 3 day after injury.
Table S4. Top 20 highest and enriched genes in each cluster.
Table S5. The subcellular locations of the top 25 character genes in IFNRM cluster.

## References

[1] Almada AE , Wagers AJ . Molecular circuitry of stem cell fate in skeletal muscle regeneration, ageing and disease. Nat Rev Mol Cell Biol 2016;17:267–279. 26956195 10.1038/nrm.2016.7 PMC4918817

[2] Blau HM , Cosgrove BD , Ho AT . The central role of muscle stem cells in regenerative failure with aging. Nat Med 2015;21:854–862. 26248268 10.1038/nm.3918 PMC4731230

[3] Brack AS , Rando TA . Tissue‐specific stem cells: lessons from the skeletal muscle satellite cell. Cell Stem Cell 2012;10:504–514. 22560074 10.1016/j.stem.2012.04.001 PMC3348769

[4] Charge SB , Rudnicki MA . Cellular and molecular regulation of muscle regeneration. Physiol Rev 2004;84:209–238. 14715915 10.1152/physrev.00019.2003

[5] Tierney MT , Stec MJ , Rulands S , Simons BD , Sacco A . Muscle stem cells exhibit distinct clonal dynamics in response to tissue repair and homeostatic aging. Cell Stem Cell 2018;22:119–127, e113. 29249462 10.1016/j.stem.2017.11.009 PMC5945549

[6] Sousa‐Victor P , Gutarra S , Garcia‐Prat L , Rodriguez‐Ubreva J , Ortet L , Ruiz‐Bonilla V , et al. Geriatric muscle stem cells switch reversible quiescence into senescence. Nature 2014;506:316–321. 24522534 10.1038/nature13013

[7] Bernet JD , Doles JD , Hall JK , Kelly Tanaka K , Carter TA , Olwin BB . p38 MAPK signaling underlies a cell‐autonomous loss of stem cell self‐renewal in skeletal muscle of aged mice. Nat Med 2014;20:265–271. 24531379 10.1038/nm.3465 PMC4070883

[8] Tidball JG . Regulation of muscle growth and regeneration by the immune system. Nat Rev Immunol 2017;17:165–178. 28163303 10.1038/nri.2016.150 PMC5452982

[9] Arnold L , Henry A , Poron F , Baba‐Amer Y , van Rooijen N , Plonquet A , et al. Inflammatory monocytes recruited after skeletal muscle injury switch into antiinflammatory macrophages to support myogenesis. J Exp Med 2007;204:1057–1069. 17485518 10.1084/jem.20070075 PMC2118577

[10] Tonkin J , Temmerman L , Sampson RD , Gallego‐Colon E , Barberi L , Bilbao D , et al. Monocyte/macrophage‐derived IGF‐1 orchestrates murine skeletal muscle regeneration and modulates autocrine polarization. Mol therapy: the j Amer Soc Gene The 2015;23:1189–1200. 10.1038/mt.2015.66 PMC4817788 25896247

[11] Mounier R , Theret M , Arnold L , Cuvellier S , Bultot L , Goransson O , et al. AMPKalpha1 regulates macrophage skewing at the time of resolution of inflammation during skeletal muscle regeneration. Cell Metab 2013;18:251–264. 23931756 10.1016/j.cmet.2013.06.017

[12] Panduro M , Benoist C , Mathis D . Treg cells limit IFN‐gamma production to control macrophage accrual and phenotype during skeletal muscle regeneration. Proc Natl Acad Sci U S A 2018;115:E2585–E2593. 29476012 10.1073/pnas.1800618115 PMC5856564

[13] Kuswanto W , Burzyn D , Panduro M , Wang KK , Jang YC , Wagers AJ , et al. Poor repair of skeletal muscle in aging mice reflects a defect in local, interleukin‐33‐dependent accumulation of regulatory T cells. Immunity 2016;44:355–367. 26872699 10.1016/j.immuni.2016.01.009 PMC4764071

[14] Patsalos A , Simandi Z , Hays TT , Peloquin M , Hajian M , Restrepo I , et al. In vivo GDF3 administration abrogates aging related muscle regeneration delay following acute sterile injury. Aging Cell 2018;17:e12815. 30003692 10.1111/acel.12815 PMC6156497

[15] Joe AW , Yi L , Natarajan A , Le Grand F , So L , Wang J , et al. Muscle injury activates resident fibro/adipogenic progenitors that facilitate myogenesis. Nat Cell Biol 2010;12:153–163. 20081841 10.1038/ncb2015 PMC4580288

[16] Lemos DR , Babaeijandaghi F , Low M , Chang CK , Lee ST , Fiore D , et al. Nilotinib reduces muscle fibrosis in chronic muscle injury by promoting TNF‐mediated apoptosis of fibro/adipogenic progenitors. Nat Med 2015;21:786–794. 26053624 10.1038/nm.3869

[17] Lukjanenko L , Jung MJ , Hegde N , Perruisseau‐Carrier C , Migliavacca E , Rozo M , et al. Loss of fibronectin from the aged stem cell niche affects the regenerative capacity of skeletal muscle in mice. Nat Med 2016;22:897–905. 27376579 10.1038/nm.4126 PMC5467443

[18] Liu L , Charville GW , Cheung TH , Yoo B , Santos PJ , Schroeder M , et al. Impaired notch signaling leads to a decrease in p53 activity and mitotic catastrophe in aged muscle stem cells. Cell Stem Cell 2018;23:544–556, e544. 30244867 10.1016/j.stem.2018.08.019 PMC6173623

[19] Lukjanenko L , Karaz S , Stuelsatz P , Gurriaran‐Rodriguez U , Michaud J , Dammone G , et al. Aging disrupts muscle stem cell function by impairing matricellular WISP1 secretion from fibro‐adipogenic progenitors. Cell Stem Cell 2019;24:433–446, e437. 30686765 10.1016/j.stem.2018.12.014 PMC6408230

[20] Zhang C , Wang C , Li Y , Miwa T , Liu C , Cui W , et al. Complement C3a signaling facilitates skeletal muscle regeneration by regulating monocyte function and trafficking. Nat Commun 2017;8:2078. 29233958 10.1038/s41467-017-01526-z PMC5727192

[21] Zhang L , Dong Y , Dong Y , Cheng J , Du J . Role of integrin‐beta3 protein in macrophage polarization and regeneration of injured muscle. J Biol Chem 2012;287:6177–6186. 22210777 10.1074/jbc.M111.292649 PMC3307266

[22] Varga T , Mounier R , Patsalos A , Gogolak P , Peloquin M , Horvath A , et al. Macrophage PPARgamma, a lipid activated transcription factor controls the growth factor GDF3 and skeletal muscle regeneration. Immunity 2016;45:1038–1051. 27836432 10.1016/j.immuni.2016.10.016 PMC5142832

[23] Giannakis N , Sansbury BE , Patsalos A , Hays TT , Riley CO , Han X , et al. Dynamic changes to lipid mediators support transitions among macrophage subtypes during muscle regeneration. Nat Immunol 2019;20:626–636. 30936495 10.1038/s41590-019-0356-7 PMC6537107

[24] Xu C , Zhang C , Ji J , Wang C , Yang J , Geng B , et al. CD36 deficiency attenuates immune‐mediated hepatitis in mice by modulating the proapoptotic effects of CXC chemokine ligand 10. Hepatology 2018;67:1943–1955. 29220536 10.1002/hep.29716

[25] Wang HJ , Zhou Y , Liu RM , Qin YS , Cen YH , Hu LY , et al. IP‐10/CXCR3 axis promotes the proliferation of vascular smooth muscle cells through ERK1/2/CREB signaling pathway. Cell Biochem Biophys 2017;75:139–147. 28111710 10.1007/s12013-017-0782-9

[26] Gao J , Wu L , Wang Y , Cui S , Duan S , Dong Z , et al. Knockdown of CXCL10 inhibits mesangial cell proliferation in murine habu nephritis via ERK signaling. Cell Physiol Biochem 2017;42:2118–2129. 28810249 10.1159/000479914

[27] Bodnar RJ , Yates CC , Wells A . IP‐10 blocks vascular endothelial growth factor‐induced endothelial cell motility and tube formation via inhibition of calpain. Circ Res 2006;98:617–625. 16484616 10.1161/01.RES.0000209968.66606.10 PMC3826264

[28] Ren H , Accili D , Duan C . Hypoxia converts the myogenic action of insulin‐like growth factors into mitogenic action by differentially regulating multiple signaling pathways. Proc Natl Acad Sci U S A 2010;107:5857–5862. 20231451 10.1073/pnas.0909570107 PMC2851893

[29] Keren A , Tamir Y , Bengal E . The p38 MAPK signaling pathway: a major regulator of skeletal muscle development. Mol Cell Endocrinol 2006;252:224–230. 16644098 10.1016/j.mce.2006.03.017

[30] Du J , Wang X , Miereles C , Bailey JL , Debigare R , Zheng B , et al. Activation of caspase‐3 is an initial step triggering accelerated muscle proteolysis in catabolic conditions. J Clin Invest 2004;113:115–123. 14702115 10.1172/JCI200418330 PMC300763

[31] Xie D , McElhaney JE . Lower GrB+ CD62Lhigh CD8 TCM effector lymphocyte response to influenza virus in older adults is associated with increased CD28null CD8 T lymphocytes. Mech Ageing Dev 2007;128:392–400. 17570460 10.1016/j.mad.2007.05.001 PMC2169430

[32] Albert EJ , Marshall JS . Aging in the absence of TLR2 is associated with reduced IFN‐gamma responses in the large intestine and increased severity of induced colitis. J Leukoc Biol 2008;83:833–842. 18223102 10.1189/jlb.0807557

[33] Cheng M , Nguyen MH , Fantuzzi G , Koh TJ . Endogenous interferon‐gamma is required for efficient skeletal muscle regeneration. Am J Physiol Cell Physiol 2008;294:C1183–C1191. 18353892 10.1152/ajpcell.00568.2007

[34] Zhang J , Xiao Z , Qu C , Cui W , Wang X , Du J . CD8 T cells are involved in skeletal muscle regeneration through facilitating MCP‐1 secretion and Gr1(high) macrophage infiltration. J Immunol 2014;193:5149–5160. 25339660 10.4049/jimmunol.1303486

[35] Foster W , Li Y , Usas A , Somogyi G , Huard J . Gamma interferon as an antifibrosis agent in skeletal muscle. J orthop resea: official publica Orthop Res Soc 2003;21:798–804. 10.1016/S0736-0266(03)00059-7 12919866

[36] Villalta SA , Deng B , Rinaldi C , Wehling‐Henricks M , Tidball JG . IFN‐gamma promotes muscle damage in the mdx mouse model of Duchenne muscular dystrophy by suppressing M2 macrophage activation and inhibiting muscle cell proliferation. J Immunol 2011;187:5419–5428. 22013114 10.4049/jimmunol.1101267 PMC3208069

[37] Cheung WW , Paik KH , Mak RH . Inflammation and cachexia in chronic kidney disease. Pediatr Nephrol 2010;25:711–724. 20111974 10.1007/s00467-009-1427-z

[38] Yoshida T , Galvez S , Tiwari S , Rezk BM , Semprun‐Prieto L , Higashi Y , et al. Angiotensin II inhibits satellite cell proliferation and prevents skeletal muscle regeneration. J Biol Chem 2013;288:23823–23832. 23831688 10.1074/jbc.M112.449074 PMC3745329

[39] Wang H , Melton DW , Porter L , Sarwar ZU , McManus LM , Shireman PK . Altered macrophage phenotype transition impairs skeletal muscle regeneration. Am J Pathol 2014;184:1167–1184. 24525152 10.1016/j.ajpath.2013.12.020 PMC3969996

[40] Ivashkiv LB . IFNgamma: signalling, epigenetics and roles in immunity, metabolism, disease and cancer immunotherapy. Nat Rev Immunol 2018;18:545–558. 29921905 10.1038/s41577-018-0029-z PMC6340644

[41] Hu X , Ivashkiv LB . Cross‐regulation of signaling pathways by interferon‐gamma: implications for immune responses and autoimmune diseases. Immunity 2009;31:539–550. 19833085 10.1016/j.immuni.2009.09.002 PMC2774226

[42] Shibahara T , Wilcox JN , Couse T , Madara JL . Characterization of epithelial chemoattractants for human intestinal intraepithelial lymphocytes. Gastroenterology 2001;120:60–70. 11208714 10.1053/gast.2001.20904

[43] Salomon I , Netzer N , Wildbaum G , Schif‐Zuck S , Maor G , Karin N . Targeting the function of IFN‐gamma‐inducible protein 10 suppresses ongoing adjuvant arthritis. J Immunol 2002;169:2685–2693. 12193742 10.4049/jimmunol.169.5.2685

[44] Chan CC , Cheng LY , Lin CL , Huang YH , Lin HC , Lee FY . The protective role of natural phytoalexin resveratrol on inflammation, fibrosis and regeneration in cholestatic liver injury. Mol Nutr Food Res 2011;55:1841–1849. 22086758 10.1002/mnfr.201100374

[45] Shin SD , Shin A , Mayagoitia K , Wilson CG , Bellinger DL , Soriano S . Interferon downstream signaling is activated early in pre‐symptomatic Niemann‐Pick disease type C. Neurosci Lett 2019;706:43–50. 31067492 10.1016/j.neulet.2019.05.005

[46] Skripuletz T , Hackstette D , Bauer K , Gudi V , Pul R , Voss E , et al. Astrocytes regulate myelin clearance through recruitment of microglia during cuprizone‐induced demyelination. Brain: a j neur 2013;136:147–167. 10.1093/brain/aws262 23266461

[47] Deyhle MR , Hafen PS , Parmley J , Preece CN , Robison M , Sorensen JR , et al. CXCL10 increases in human skeletal muscle following damage but is not necessary for muscle regeneration. Physiol Rep 2018;6:e13689. 29696819 10.14814/phy2.13689 PMC5917067

[48] von Haehling S , Morley JE , Coats AJS , Anker SD . Ethical guidelines for publishing in the *Journal of Cachexia, Sarcopenia and Muscle*: update. J cachexia, sarcop and muscle 2019 2019;10:1143–1145. 10.1002/jcsm.12501 PMC6818444 31661195

