# Supplementary material for: Age‐related decline of interferon‐gamma responses in macrophage impairs satellite cell proliferation and regeneration
Source: J Cachexia Sarcopenia Muscle. 2020 Jul 29;11(5):1291–305. doi: 10.1002/jcsm.12584 (PMC7567146; doi:10.1002/jcsm.12584)
Supplement: Supplementary file 1 — Figure S1. IFN‐response pathway was downregulated in aged muscle after injury. Figure S1. IFN‐response pathway was downregulated in aged muscle after injury. Figure S3. Distribution of marker genes in cell cluster of muscle at 3 day after injury. Figure S4. The flow scheme of identify the IFNRM cluster from monocyte/macrophage cluster by Loupe Cell software. Figure S5. Recombinant CXCL10 promotes the differentiation of primary MuSCs in vitro. Figure S6. Recombinant CXCL10 treatment decreases the muscle fibrosis during regeneration in aged mice. Table S1. Antibody list. Table S2. Oligonucleotide sequences for quantitative realtime‐PCR. Table S3. Expression levels of IFN‐responsive genes in muscle from 0 day and 3 day after injury. Table S4. Top 20 highest and enriched genes in each cluster. Table S5. The subcellular locations of the top 25 character genes in IFNRM cluster. [file JCSM-11-1291-s001.docx]

**Supplementary information**

**Age-related decline of IFN-gamma responses in macrophage impairs satellite cell proliferation and regeneration**

Congcong Zhang, Naixuan Cheng, Bokang Qiao, Fan Zhang, Jian Wu, Chang Liu, Yulin Li, Jie Du^*^


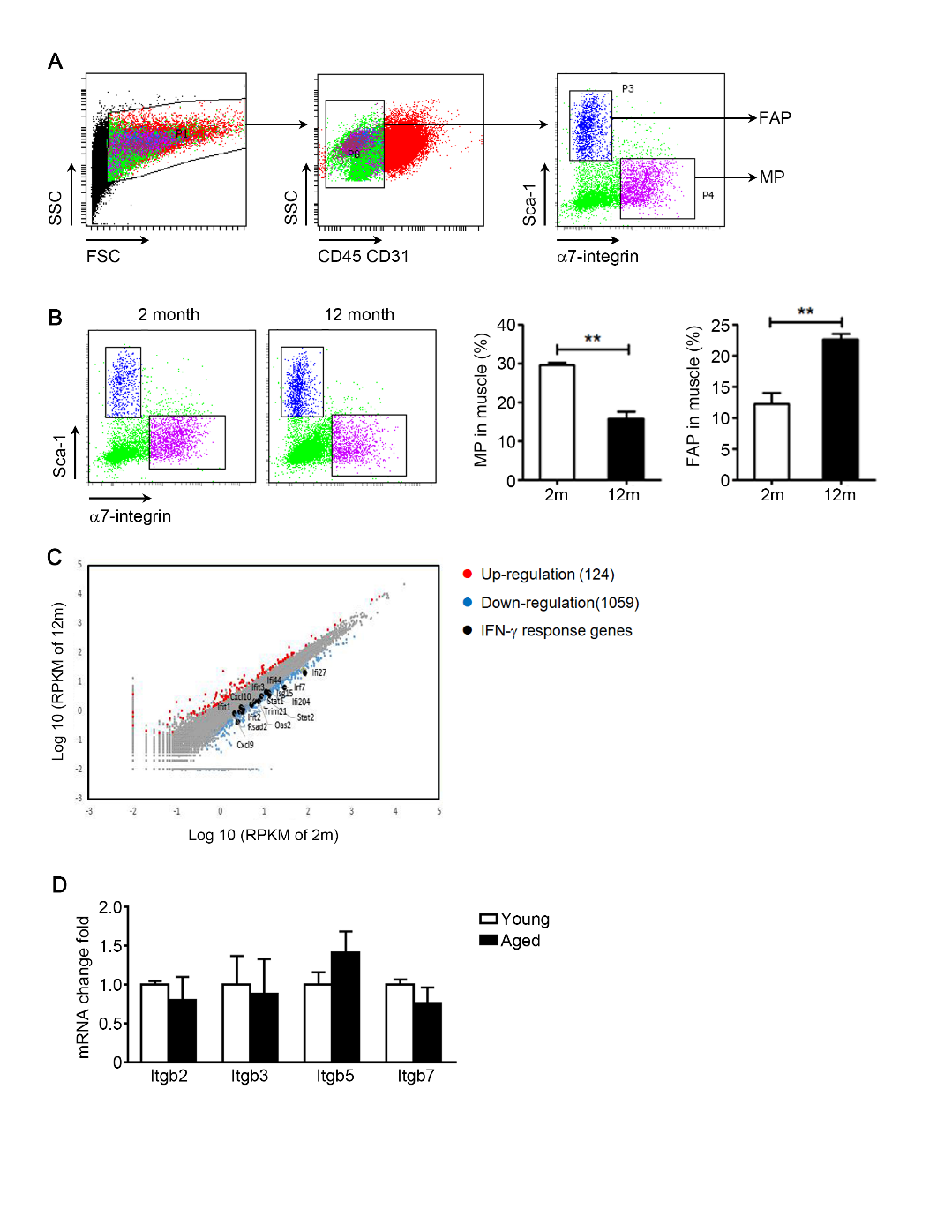


**Figure S1. IFN-response pathway was downregulated in aged muscle after injury.**

(A) The gating strategy of CD45^-^CD31^-^α7-integrin^+^ myoblast (MPs) and CD45^-^CD31^-^Sca-1^+^ FAPs in muscle. (B) At day 5 after injury, the number of MPs and FAPs in muscles from 2-months and 12-months WT mice were detected by FACS. The right graph indicated the percentages of MP and FAP in each group (n=4 per group). (C) Scatterplot of RNA-seq expression data from injured 2-months and 12-months WT muscles at day 5 after CTX injection. Different expression genes were filtered by using a threshold of twofold change and false discovery rate (FDR) < 0.001. Red dots indicated up-regulated genes, Blue dots indicated down-regulated genes, black dots indicated genes associated with response to IFN-γ. (D) The mRNA level of Integrins in muscles from 2-months and 24-months WT mice were assessed by realtime-PCR at day 5 after injury (n=4 per group).

Data represent the mean ± SEM. **P < 0.01, by two-tailed Student’s t-test.


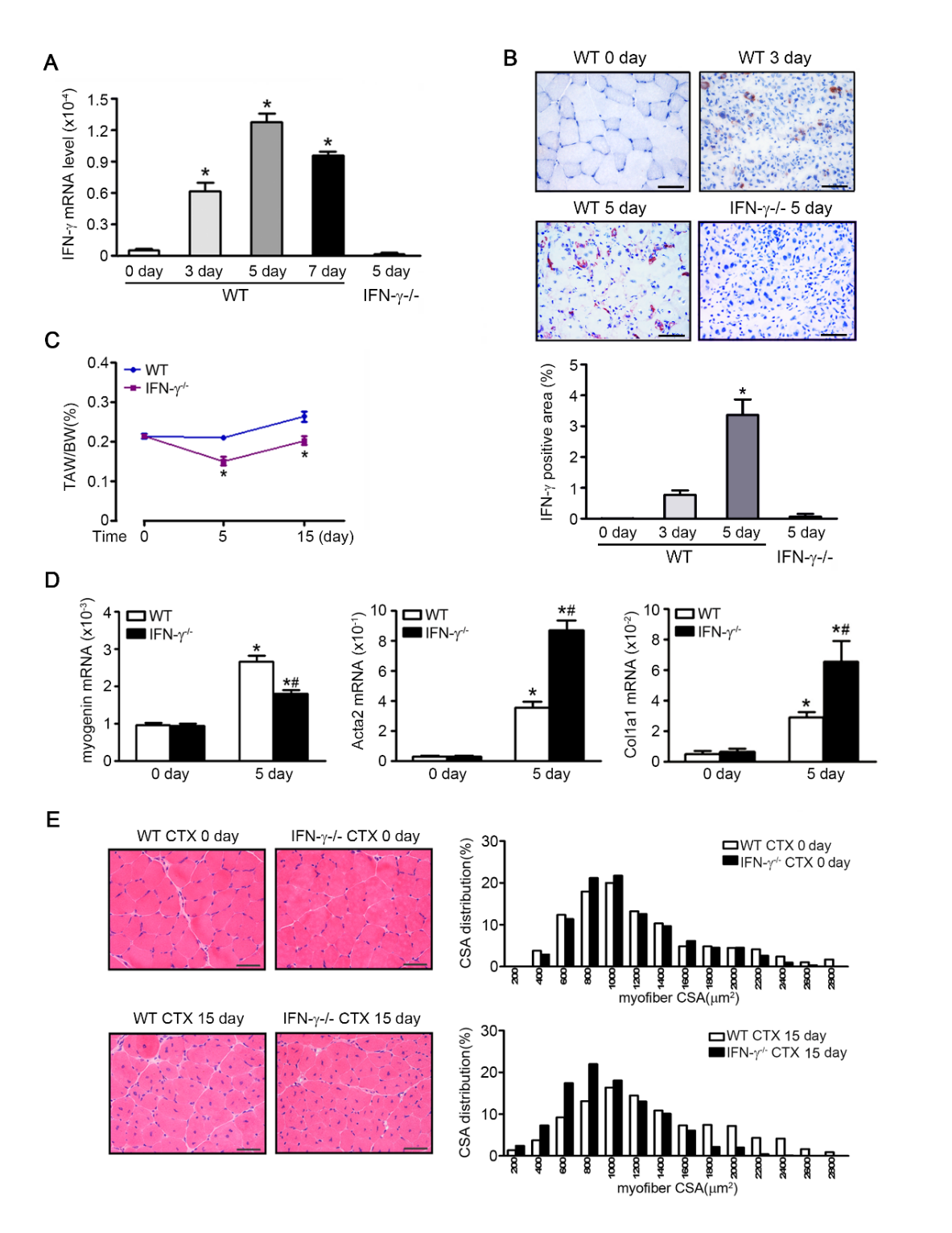


**Figure S2. IFN-gamma deficiency impairs muscle regeneration.**

(A) Time sequence for IFN-γ mRNA expression in injured TA muscle was accessed by realtime-PCR. Values were corrected by GAPDH (n=3 per group). ***P < 0.05, by one way-ANOVA.** (B) IFN-γ expression was detected by IHC staining with anti-IFN-γ antibody (brown) in injured TA muscle (Bars=50 μm, 3 per group). (C) The ratio of TA weight (TAW) to body weight (BW) of WT and IFN-γ-/- mice at different timepoint after injury (n=6 per group). (D) The mRNA level of Myogenin, Acta2, Col1a1 in muscles from WT and IFN-γ-/- mice were assessed by realtime-PCR at day 5 after injury (n=4 per group). (E) Left: HE staining of TA muscle from WT and IFN-γ-/- mice at 0 day and 15 day after injury (Bars=50 μm). Right: Quantification of the cross-section distribution of newly formed myofiber based on HE staining (n=4 per group).

Data represent the mean ± SEM. *P< 0.05 compared with WT 0 day, # P< 0.05 compared with WT 5 day, by two-tailed Student’s t-test.


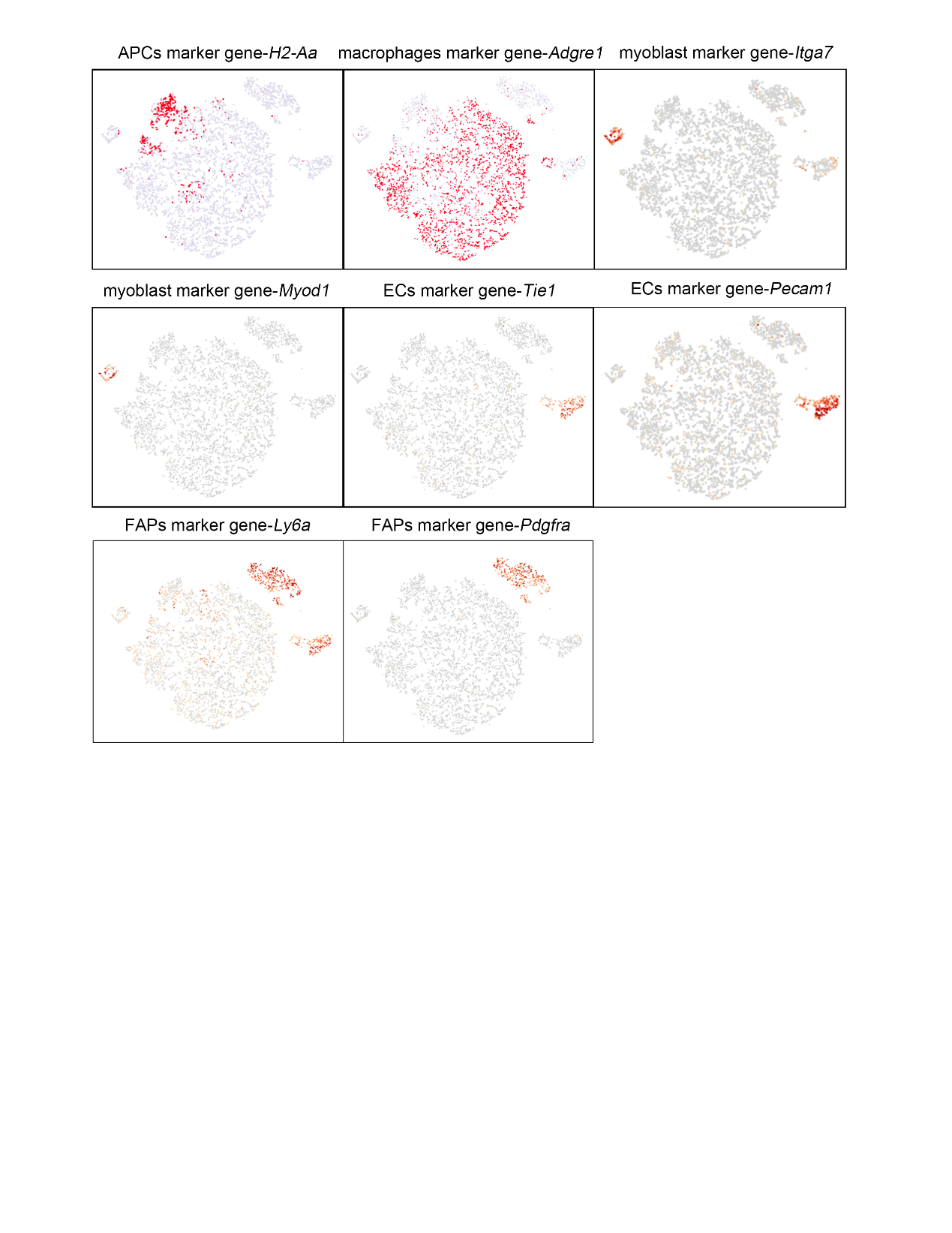


**Figure S3. Distribution of marker genes in cell cluster of muscle at 3 day after injury.**

t-SNE visualization overlaid with the average expression (UMI) of known marker genes of various cells in the single-cell RNA-seq data. Red indicates the high average UMI, grey indicates the low average UMI.


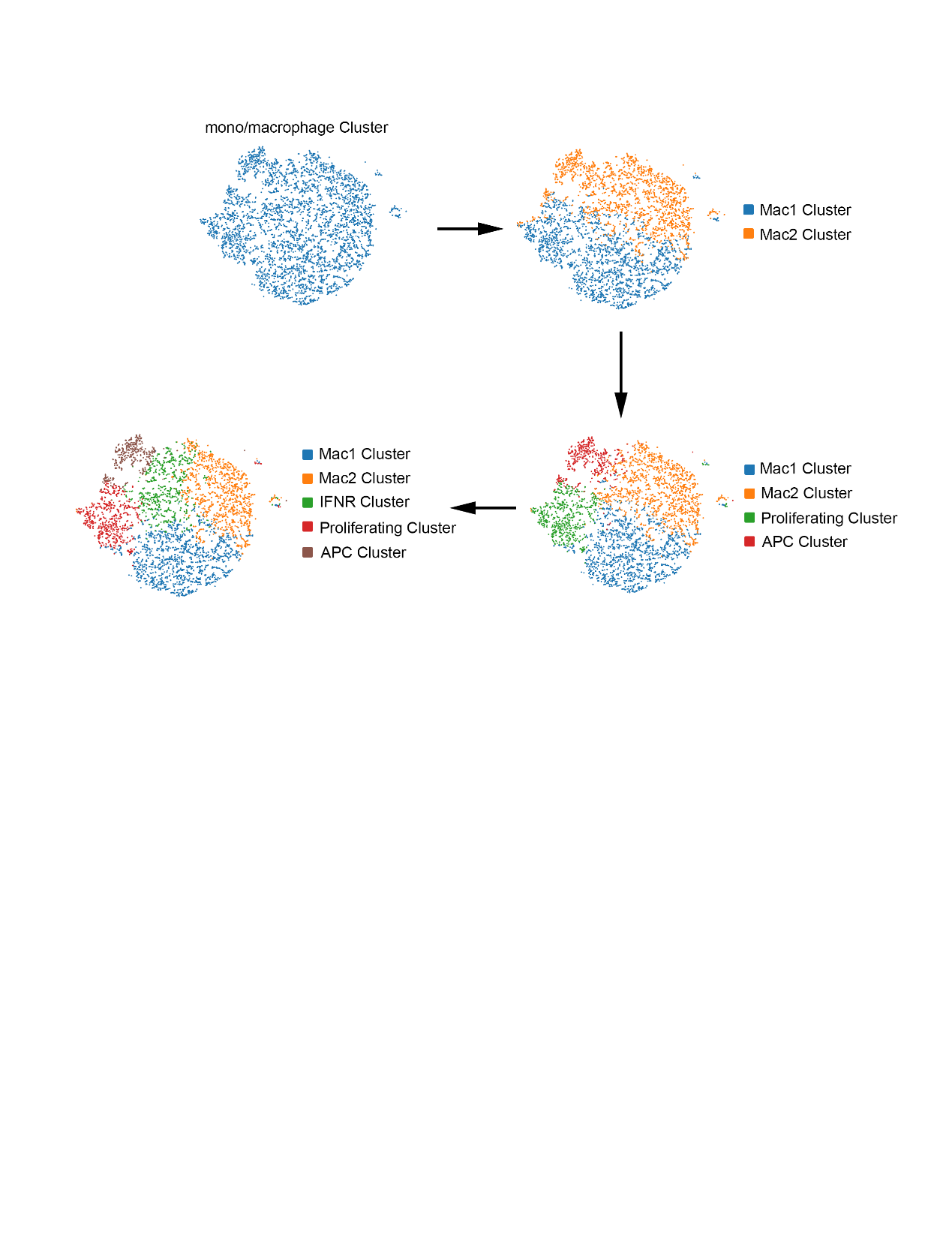


Figure S4. The flow scheme of identify the IFNRM cluster from monocyte/macrophage cluster by Loupe Cell software.


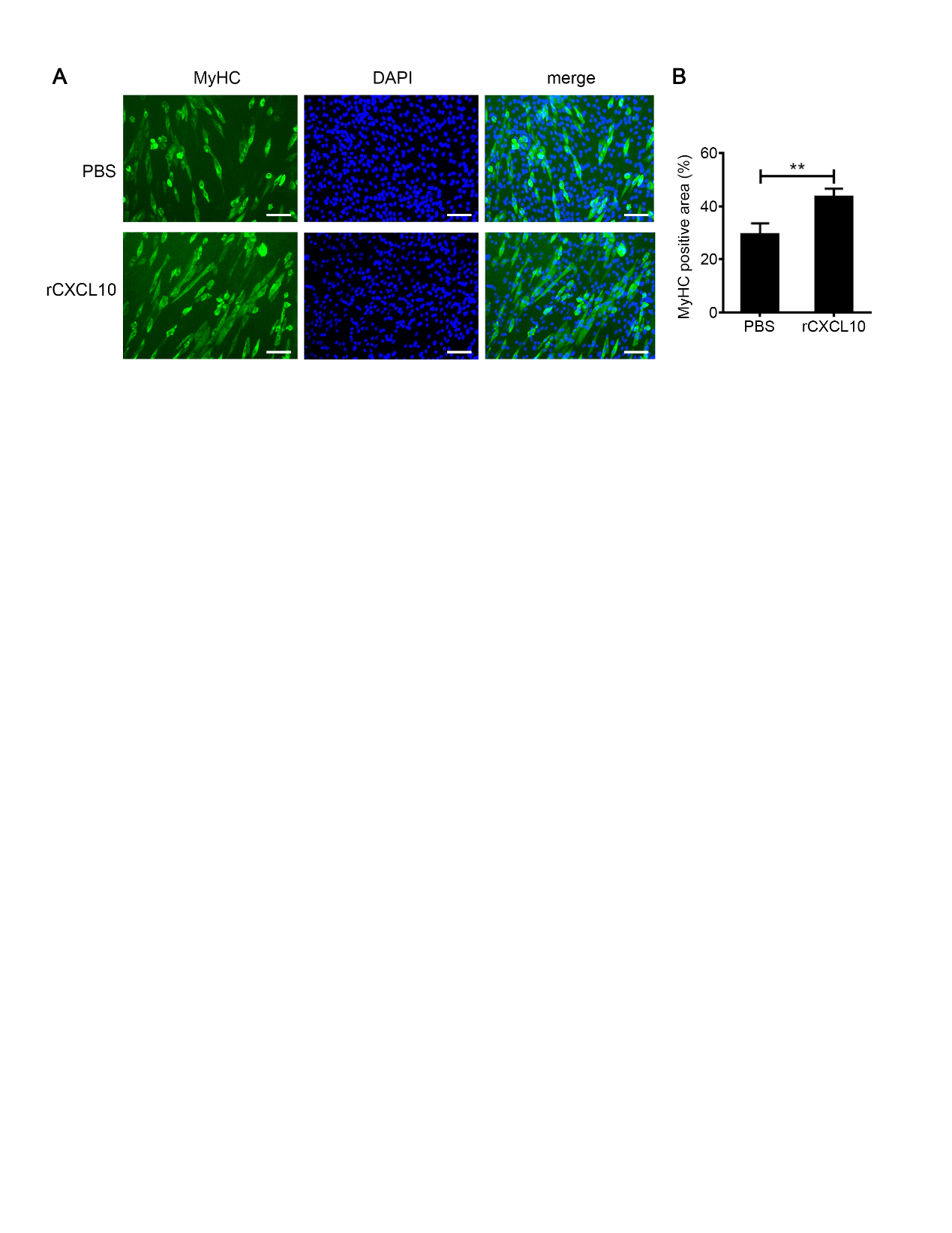


**Figure S5. Recombinant CXCL10 promotes the differentiation of primary MuSCs in vitro.**

(A) The primary myoblasts were cultured in differentiation medium with either PBS or rCXCL10 (20ng/ml) for 3 days, and myogenic differentiation was assessed by immunostaining against MyHC (green) (Bars=50 μm). (B) The fusion index was calculated by the MyHC positive area per field (n=6 per group). Data represent the mean ± SEM. **P < 0.01, by two-tailed Student’s t-test.


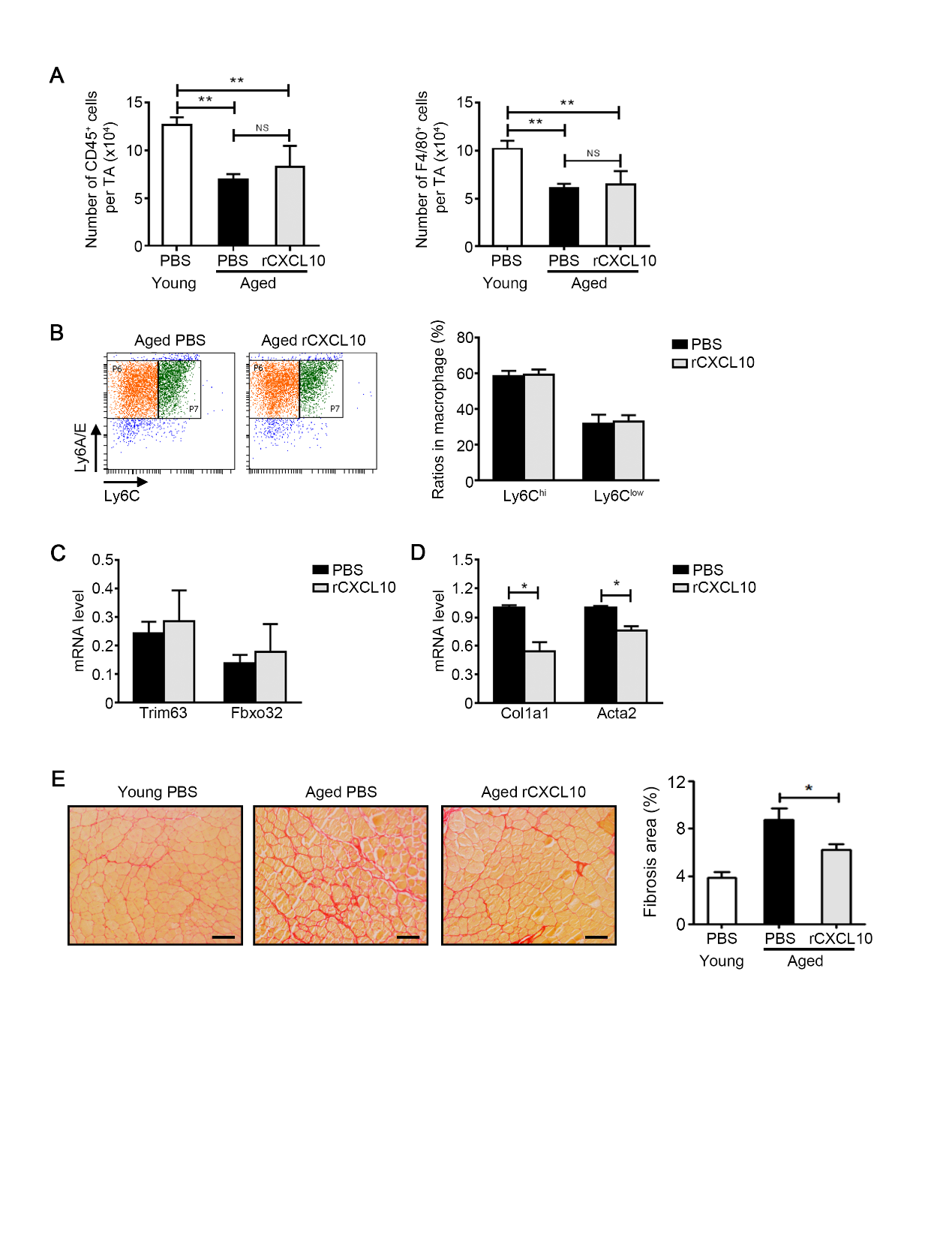
**Figure S6. Recombinant CXCL10 treatment decreases the muscle fibrosis during regeneration in aged mice.**

(A) The number of CD45^+^ leukocytes and CD45^+^F4/80^+^ macrophages per TA muscle from PBS or rCXCL10-treated aged mouse were accessed by flow cytometry at 3 day after injury (n=4 per group). (B)The ratios of Ly6E^+^Ly6C^hi^ and Ly6E^+^Ly6C^low^ cells in macrophages were accessed by flow cytometry at 3 day after injury (n=4 per group). (C) The mRNA levels of Trim63, Fbxo32 in PBS or rCXCL10-treated aged muscles at 5 day after injury was accessed by realtime-PCR (n=4 per group). (D) the mRNA levels of Col1a1, Acta2 in PBS or rCXCL10-treated aged muscles at 5 day after injury was accessed by realtime-PCR (n=4 per group). (E) The ratio of fibrosis area in PBS or rCXCL10-treated aged muscles was accessed by Sirus Red staining at 15 day after injury (Bars=100 μm, n=4 per group).

Data represent the mean ± SEM. *P < 0.05, by two-tailed Student’s t-test.

**Table S1. Antibody list.**

| **Antibodies** | **Isotype** | **Dilution** | **Source** | **Identifier** |
| --- | --- | --- | --- | --- |
| **IHC and IF antibodies** | | | | |
| WGA | Mouse monoclonal | 1:200 | Sigma | Cat#L4895 |
| Laminin | Rabbit polyclonal | 1:100 | Abcam | Cat#ab11575 |
| IFN-γ | Rat monoclonal | 1:50 | Abcam | Cat#ab23637 |
| Ki-67 | Rabbit polyclonal | 1:100 | Abcam | Cat#Ab15580 |
| Pax7 | Mouse monoclonal | 1:200 | DSHB | RRID: AB_528428 |
| MyHC(MF-20) | Mouse monoclonal | 1:200 | DSHB | RRID:AB_2147781 |
| anti-rat IgG Alexa 488 | Goat polyclonal | 1:1000 | Cell Signaling Technology | Cat# 4416 |
| anti-rabbit IgG Alexa 488 | Goat polyclonal | 1:1000 | Invitrogen | Cat# A-11008 |
| anti-mouse IgG Alexa 555 | Goat polyclonal | 1:1000 | Invitrogen | Cat# A-21422 |
| anti-rabbit IgG Alexa 555 | Goat polyclonal | 1:1000 | Invitrogen | Cat# A-21428 |
| anti-mouse IgG Alexa 488 | Goat polyclonal | 1:1000 | Invitrogen | Cat# A-11001 |
| **Flow cytometry antibody** | | | | |
| CD45 PerCP-Cy5.5 | Rat anti mouse | 1:100 | BD | Cat#550994 |
| CD11b APC-Cy7 | Rat anti mouse | 1:100 | BD | Cat#557657 |
| F4/80 PE | Rat anti mouse | 1:100 | eBioscience | Cat#12-4801-83 |
| F4/80 APC | Rat anti mouse | 1:100 | eBioscience | Cat#17-4801-82 |
| Gr1 FITC | Rat anti mouse | 1:100 | Biolegend | Cat#108406 |
| CD3e PE-CF594 | Rat anti mouse | 1:100 | BD | Cat#562286 |
| CD4 PE-Cy7 | Rat anti mouse | 1:100 | eBioscience | Cat#25-0041-82 |
| CD8a APC | Rat anti mouse | 1:100 | eBioscience | Cat#17-0081-82 |
| IFN-γ Alexa 488 | Rat anti mouse | 1:100 | Biolegend | Cat#508513 |
| Ly6C V450 | Rat anti mouse | 1:100 | BD | Cat#560594 |
| CD183(CXCR3) BV421 | Hamster anti mouse | 1:100 | BD | Cat#562937 |
| CD45 FITC | Rat anti mouse | 1:100 | BD | Cat#553079 |
| Ly6A/E PE-cy7 | Rat anti mouse | 1:100 | BD | Cat#558162 |
| CD31 FITC | Rat anti mouse | 1:100 | BD | Cat#553372 |
| α7-integrin APC | Mouse monoclonal | 1:10 | RD | Cat#FAB3518A |
| **Western Blot antibodies** | | | | |
| p-ERK1/2 | Rabbit polyclonal | 1:1000 | Cell Signaling Technology | Cat# 4370 |
| ERK1/2 | Rabbit polyclonal | 1:1000 | Cell Signaling Technology | Cat# 4695 |
| p-MAPKp38 | Rabbit polyclonal | 1:1000 | Cell Signaling Technology | Cat# 4511 |
| MAPKp38 | Rabbit polyclonal | 1:1000 | Cell Signaling Technology | Cat# 8690 |
| GAPDH | Mouse monoclonal | 1:2000 | Cell Signaling Technology | Cat# 2118 |

**Table S2. Oligonucleotide sequences for quantitative realtime-PCR.**

| Gene | Forward 5’­3’ | Reward 5’­3’ |
| --- | --- | --- |
| Ifng | GCTTTAACAGCAGGCCAGAC | GCACCAGGTGTCAAGTCTCT |
| Irf1 | ATCTCGGGCATCTTTCGCTT | TGCATCTCTAGCCAGGGTCT |
| Irf7 | GGGACCTCTTGCTTCAGGTT | AGGGTTCCTCGTAAACACGG |
| Ifit1 | TCCGTAGGAAACATCGCGTA | TGTTGCTTGTAGCAGAGCCC |
| Ifit2 | AGATGAGACTTAGAGGTGCTGC | TGTGTCAAAGCGCTCAAAGC |
| Ifit3 | CTGAACTGCTCAGCCCACA | TTCCCGGTTGACCTCACTCA |
| Cxcl10 | ATGACGGGCCAGTGAGAATG | AGGCTCGCAGGGATGATTTC |
| Myod1 | AGCATAGTGGAGCGCATCTC | GGTCTGGGTTCCCTGTTCTG |
| Myog | CAGCCCAGCGAGGGAATTTA | AGAAGCTCCTGAGTTTGCCC |
| Acta2 | TAACCCTTCAGCGTTCAGCC | ACATAGCTGGAGCAGCGTCT |
| Col1a1 | CGATGGATTCCCGTTCGAGT | CATTAGGCGCAGGAAGGTCA |
| Ly6c2 | ACCCTTCTCTGAGGATGGACA | GCTGGGCAGGAAGTCTCAAT |
| Isg15 | CGATTTCCTGGTGTCCGTGA | AGACCCAGACTGGAAAGGGT |
| Rsad2 | ATTCTGGATGTTGGCGTGGA | ATACTTTCCGCCACGCTTCA |
| Usp18 | CAGGAGTCCCTGATTTGCGT | GGGCTGGACGAAACATCTCA |
| Itgb2 | TGCCGCATTCAATGTGACTTT | CTTCTTGACGTTGTTGAGGTCAT |
| Itgb3 | CCACACGAGGCGTGAACTC | CTTCAGGTTACATCGGGGTGA |
| Itgb5 | GCCAAGTTCCAAAGCCTCAA | AGGCGAAATCGACAGTGTGT |
| Itgb7 | ACCTGAGCTACTCAATGAAGGA | CACCGTTTTGTCCACGAAGG |
| Trim63 | GTGTGAGGTGCCTACTTGCTC | GCTCAGTCTTCTGTCCTTGGA |
| Fbxo32 | CAGCTTCGTGAGCGACCTC | GGCAGTCGAGAAGTCCAGTC |
| Actb | GCAAGCAGGAGTACGATGAGT | AACGCAGCTCAGTAACAGTC |
| Gapdh | GCAAGCAGGAGTACGATGAGT | AACGCAGCTCAGTAACAGTC |

**Table S3. Expression levels of IFN-responsive genes in muscle from 0 day and 3 day after injury.**

| Gene  Symbol | WT0D-Expression | WT3D-Expression | log2FC  (WT3D/WT0D) | P-value |
| --- | --- | --- | --- | --- |
| Tlr7 | 0.01 | 9.33 | 9.865733 | 1.07E-206 |
| Runx3 | 0.01 | 5.76 | 9.169925 | 1.13E-117 |
| Aif1 | 0.31 | 78.54 | 7.985016 | 0 |
| Cxcl16 | 0.24 | 58.6 | 7.931722 | 0 |
| Tlr8 | 0.04 | 8.71 | 7.766529 | 1.81E-215 |
| Gbp8 | 0.01 | 1.35 | 7.076816 | 2.26E-21 |
| Ifit1bl1 | 0.01 | 1.31 | 7.033423 | 2.67E-17 |
| Cxcl10 | 0.15 | 18.91 | 6.978043 | 7.34E-114 |
| Isg15 | 0.74 | 43.87 | 5.889566 | 6.43E-166 |
| Runx1 | 0.34 | 18.66 | 5.77827 | 0 |
| Stat4 | 0.01 | 0.51 | 5.672425 | 1.43E-08 |
| Arg2 | 0.01 | 0.47 | 5.554589 | 0.000169 |
| Ticam2 | 0.05 | 2.34 | 5.548437 | 8.67E-40 |
| Irf7 | 2.51 | 112.38 | 5.484554 | 0 |
| Acod1 | 0.01 | 0.35 | 5.129283 | 5.93E-06 |
| Ciita | 0.1 | 3.32 | 5.053111 | 1.26E-102 |
| Socs1 | 0.31 | 8.85 | 4.835337 | 9.74E-53 |
| Ifit3 | 1.89 | 51.79 | 4.776215 | 0 |
| Ifit1 | 0.5 | 11.73 | 4.552131 | 2.90E-141 |
| Bst2 | 10 | 222.22 | 4.473917 | 0 |
| Irf8 | 2.2 | 44 | 4.321928 | 0 |
| Ifit2 | 0.89 | 17.1 | 4.264047 | 3.67E-289 |
| Ifitm3 | 32.82 | 397.53 | 3.598417 | 0 |
| Tlr4 | 0.32 | 3.44 | 3.426265 | 5.81E-102 |
| Ulbp1 | 0.11 | 0.93 | 3.079727 | 4.73E-07 |
| Cxcl9 | 0.54 | 4.43 | 3.036275 | 4.95E-44 |
| Irf5 | 5.81 | 41.09 | 2.822177 | 7.63E-286 |
| Irf4 | 0.07 | 0.49 | 2.807355 | 8.67E-09 |
| Stat1 | 3.62 | 24.15 | 2.737962 | 2.50E-300 |
| Isg20 | 0.98 | 6.46 | 2.720681 | 2.56E-22 |
| Irf1 | 3.95 | 24.15 | 2.612099 | 1.48E-145 |
| Ifitm2 | 28.86 | 170.66 | 2.563982 | 1.35E-289 |
| Irf9 | 2.73 | 15.41 | 2.496894 | 1.82E-97 |
| Gbp2 | 2.63 | 14.16 | 2.428687 | 9.03E-92 |
| Cfh | 0.51 | 2.64 | 2.371969 | 1.87E-30 |
| Gbp3 | 2.19 | 10.72 | 2.291302 | 1.79E-69 |
| Ifit3b | 2.58 | 12.16 | 2.2367 | 1.12E-58 |
| Tlr3 | 0.52 | 2.14 | 2.041027 | 2.45E-20 |
| Gbp5 | 1.46 | 4.17 | 1.514079 | 6.54E-19 |
| Gbp7 | 2.23 | 6.2 | 1.475225 | 7.05E-48 |
| Irf3 | 9.61 | 25.69 | 1.418599 | 5.72E-50 |
| Hmgb1 | 45.44 | 120.49 | 1.406879 | 0 |
| Tgtp2 | 3.09 | 7.54 | 1.286958 | 1.51E-24 |
| Gbp9 | 3.88 | 9.46 | 1.285784 | 1.32E-57 |
| Cx3cl1 | 0.71 | 1.59 | 1.163136 | 4.83E-06 |
| Sumo1 | 30.34 | 66.94 | 1.141647 | 1.28E-73 |
| Tgtp1 | 1.33 | 2.8 | 1.074001 | 7.75E-08 |

FC, fold change; genes were filtered by log2FC >1, P-value<0.001, ranged by log2FC.

**Table S4. Top 20 highest and enriched genes in each cluster.**

| Cluster0 | Cluster1 | Cluster2 | Cluster3 | Cluster4 | Cluster6 | Cluster6 | Cluster7 |
| --- | --- | --- | --- | --- | --- | --- | --- |
| **Mac1** | **Mac2** | **IFNRM** | **Proliferating Mac** | **APC** | **FAP** | **EC** | **Myoblast** |
| Atp6v0d2 | Lpcat2 | Ly6c2 | Nusap1 | Cd7 | Thbs4 | Ccl21a | Ttn |
| Fabp5 | Sepp1 | Plac8 | Hist1h2ae | Cd209a | Sfrp1 | Tmem252 | Chrna1 |
| Il7r | Trf | F13a1 | Hist1h1b | Klrd1 | Prg4 | Cldn5 | Neb |
| Mmp12 | Apoe | Clec4e | Pbk | H2-Oa | Dpep1 | Mall | Tnnt2 |
| F7 | Ypel3 | Ms4a4c | Prc1 | Gpr171 | Ptx3 | Myct1 | Actn3 |
| Ctsl | Pla2g7 | Ccl12 | Spc25 | Amica1 | Dcn | Tinagl1 | Rbm24 |
| Lgals3 | Fcgr2b | Ccr2 | Cdk1 | Klrk1 | Scn7a | Sox18 | Cdh15 |
| Adam8 | Cx3cr1 | Ifitm6 | Ccnb1 | Bcl11a | Pdgfra | Mmrn2 | Vgll2 |
| Spp1 | Neurl3 | Marcksl1 | Hmmr | H2-DMb2 | Angptl1 | Robo4 | Myod1 |
| Uap1l1 | Ramp1 | Clec4a1 | Asf1b | Ifitm1 | Cilp | Sox17 | Actc1 |
| Cstb | Tnfaip8l2 | Mrc1 | Top2a | Rnase6 | Sfrp2 | Clic5 | Ankrd1 |
| Lpl | Clec4n | Rsad2 | Ccna2 | Ifitm6 | Clec3b | Gja4 | Myog |
| Clec4d | Tspan13 | Pyhin1 | Ube2c | Traf1 | Col1a1 | Grrp1 | Gal |
| Gpr137b | Cxcr4 | Ifit3 | Cenpe | Plbd1 | Smoc2 | Aplnr | Murc |
| Por | Lst1 | Phf11b | Birc5 | H2-Eb1 | Ms4a4d | Mecom | Gm7325 |
| Vat1 | Ms4a7 | Ccl6 | Mki67 | H2-Ab1 | 1500015O10Rik | Emcn | Jsrp1 |
| Plin2 | Cyp4f18 | Cxcl10 | 2810417H13Rik | H2-Aa | Myoc | Prex2 | Sox11 |
| Cd36 | Fcgr3 | Zbp1 | Rrm2 | Cd74 | Mfap5 | Pecam1 | Myl1 |
| Rnh1 | AB124611 | Irf7 | Tk1 | Mgl2 | Ccl11 | Erg | Ank1 |
| Syngr1 | Ier3 | Clec4n | Cenpf | Napsa | Thbs4 | Adgrl4 | Itga7 |

Analyzed by Loupe cell Browser software. Genes highlighted in red are known cell markers.

**Table S5. The subcellular locations of the top 25 character genes in IFNRM cluster.**

| Gene Symbol | Log2 FC | subcellular location |
| --- | --- | --- |
| Plac8 | 1.727162 | Nucleus |
| Ly6c2 | 1.62995 | Plasma membrane |
| Ms4a4c | 1.548242 | Integral component of membrane |
| Isg15 | 1.545867 | Cytoplasm, Extracellular ragion |
| Ifit3 | 1.217 | Cytoplasm, Mitochondrion |
| Rsad2 | 1.140562 | Mitochondrion |
| Irf7 | 1.103593 | Nucleus, Cytoplasm |
| Ifi204 | 1.091122 | Nucleus, Cytoplasm |
| Ly6e | 1.065084 | Plasma membrane |
| Ifitm3 | 1.055532 | Nucleus, Cytoplasm |
| Cxcl10 | 1.040777 | Extracellular ragion |
| Zbp1 | 1.008743 | Nucleus, Cytoplasm |
| Fcgr1 | 0.97412 | Plasma membrane |
| Mnda | 0.961344 | Nucleus |
| Ifi27l2a | 0.926303 | Integral component of membrane |
| Pyhin1 | 0.922377 | Nucleus |
| Rtp4 | 0.872133 | Cytoplasm |
| Slfn1 | 0.869316 | Nucleus |
| Mndal | 0.856725 | Nucleus |
| Cebpb | 0.840559 | Nucleus |
| Ifi203 | 0.840419 | Nucleus, Cytoplasm |
| Ccl12 | 0.825889 | Extracellular ragion, intracellular |
| Ccr2 | 0.810703 | Plasma membrane |
| Ifit2 | 0.78936 | Cytoplasm, endoplasmic retculum |
| Ms4a6b | 0.77394 | Integral component of membrane |

Ranged by Log2FC, FC, Fold change.
